# Entrainment of the Mammalian Cell Cycle by the Circadian Clock: Modeling Two Coupled Cellular Rhythms

**DOI:** 10.1371/journal.pcbi.1002516

**Published:** 2012-05-31

**Authors:** Claude Gérard, Albert Goldbeter

**Affiliations:** Faculté des Sciences, Université Libre de Bruxelles (ULB), Campus Plaine, CP 231, Brussels, Belgium; University of Auckland, New Zealand

## Abstract

The cell division cycle and the circadian clock represent two major cellular rhythms. These two periodic processes are coupled in multiple ways, given that several molecular components of the cell cycle network are controlled in a circadian manner. For example, in the network of cyclin-dependent kinases (Cdks) that governs progression along the successive phases of the cell cycle, the synthesis of the kinase Wee1, which inhibits the G2/M transition, is enhanced by the complex CLOCK-BMAL1 that plays a central role in the circadian clock network. Another component of the latter network, REV-ERBα, inhibits the synthesis of the Cdk inhibitor p21. Moreover, the synthesis of the oncogene c-Myc, which promotes G1 cyclin synthesis, is repressed by CLOCK-BMAL1. Using detailed computational models for the two networks we investigate the conditions in which the mammalian cell cycle can be entrained by the circadian clock. We show that the cell cycle can be brought to oscillate at a period of 24 h or 48 h when its autonomous period prior to coupling is in an appropriate range. The model indicates that the combination of multiple modes of coupling does not necessarily facilitate entrainment of the cell cycle by the circadian clock. Entrainment can also occur as a result of circadian variations in the level of a growth factor controlling entry into G1. Outside the range of entrainment, the coupling to the circadian clock may lead to disconnected oscillations in the cell cycle and the circadian system, or to complex oscillatory dynamics of the cell cycle in the form of endoreplication, complex periodic oscillations or chaos. The model predicts that the transition from entrainment to 24 h or 48 h might occur when the strength of coupling to the circadian clock or the level of growth factor decrease below critical values.

## Author Summary

The cell cycle and the circadian clock are two major cellular rhythms. These two periodic processes are tightly coupled through multiple regulatory interactions; several components of the cell cycle machinery are indeed controlled by the circadian network. By using detailed computational models for the cell cycle and circadian networks we investigate the conditions in which the mammalian cell cycle can be entrained by the circadian clock. We show that entrainment to a circadian period can occur when the period of the cell cycle prior to coupling is either smaller or larger than 24 h. Entrainment to 48 h can also be observed. The presence of multiple modes of coupling does not enlarge the domain of entrainment. Coupling to the circadian clock may also lead to complex oscillatory dynamics of the cell cycle in the form of endoreplication, complex periodic oscillations, or chaotic oscillations. The model predicts that entrainment of the cell cycle could also result from the circadian variation of a growth factor gating entry into G1, and that the transition from an entrained period of 24 h to 48 h might result from a decrease in coupling strength or in the level of growth factor.

## Introduction

The cell cycle and the circadian clock represent two major examples of cellular periodic behavior [1]. Experimental observations have long shown that these periodic processes are often coupled. In some photosynthetic organisms such as *Euglena* and cyanobacteria, the cell division cycle is gated by the circadian clock [2]–[5]. More recent experiments indicate that this situation is also encountered in a variety of cell types, including mammalian cells [6], [7]. The mammalian cell cycle is governed by a network of cyclin-dependent kinases (Cdks). Each phase of the cell cycle is controlled by a different cyclin/Cdk complex [8], [9]: cyclin D/Cdk4–6 and cyclin E/Cdk2 control the G1 phase and the G1/S transition, respectively; cyclin A/Cdk2 allows progression in the S phase of DNA replication, while cyclin B/Cdk1 brings about the G2/M transition.

Several links between the cell cycle and the circadian network have been uncovered in recent years. A number of components of the Cdk network are indeed controlled by the circadian clock, generally through induction of gene expression by a key transcriptional regulator of the circadian network such as the complex CLOCK-BMAL1. The latter complex can induce the transcription of the kinase Wee1 [10], which inhibits, through phosphorylation, the kinases Cdk1 and Cdk2. It can also inhibit, via the protein REV-ERBα the transcription of the Cdk inhibitor p21 [11], or repress the oncogene c-Myc that induces the expression of cyclin E [12], [13]. Such tight coupling to the circadian clock may readily lead to the entrainment of the cell cycle, which would explain why cell division often operates on a 24 h time scale. However, entrainment is only one possible outcome of the coupling between the two cellular rhythms. Besides the circadian gating of cell division, which occurs in precise conditions, complex oscillations in the Cdk network may also result from its control by the circadian clock.

We use a detailed computational model recently proposed for the Cdk network driving the mammalian cell cycle [14] to investigate the conditions in which it can be entrained through three distinct modes of coupling to the circadian clock. The question arises as to whether entrainment is facilitated when multiple modes of coupling occur concomitantly. The role of such redundancy can be addressed most conveniently through numerical simulations by determining whether the domain of entrainment in parameter space increases in the presence of more than one mode of coupling. We also address the possibility of entraining the cell cycle through circadian variations in the level of a growth factor inducing cell entry into the G1 phase. This potential mode of coupling is suggested by the observation of circadian variations in the levels of several growth factor receptors [15]–[17].

The coupling between the circadian clock and the cell cycle has been considered in previous theoretical studies. One such study coupled a minimal model for circadian rhythms with a model originally developed for the yeast cell cycle [18] in the presence of size control on cell division. An experimental and theoretical study of the cell cycle in cyanobacteria [4], [5] revealed circadian gating of cell division. The results were accounted for by an abstract model in which the speed of progression through the cell division cycle slows down during subjective circadian night. These studies, however, do not consider how the circadian gating mechanism is implemented at the molecular level. Likewise, entrainment has readily been achieved in an automaton model for the cell cycle, which does not incorporate any detail about the biochemical network that underlies the transitions between the cell cycle phases [19]. Recently, a model was proposed to account for the regulation of mammalian cell cycle progression and its gating by the circadian clock in regenerating liver [20]. All these studies generally consider but a single molecular link between the cell cycle and the circadian clock. Here, we use a more comprehensive computational model for the Cdk network driving the mammalian cell cycle to study its entrainment by the circadian clock. Because of its detailed biochemical nature, this model allows us to study the effect of multiple molecular links for coupling the cell cycle to the circadian clock. We characterize the domains of cell cycle entrainment to 24 h or 48 h periods and determine conditions for switching between these two patterns of entrainment. We assess whether circadian entrainment is facilitated by the presence of more than a single mode of coupling and determine the types of simple or complex oscillatory dynamics that may occur outside the domain of entrainment.

## Results

### Oscillatory dynamics of the Cdk network

**Figure 1:**
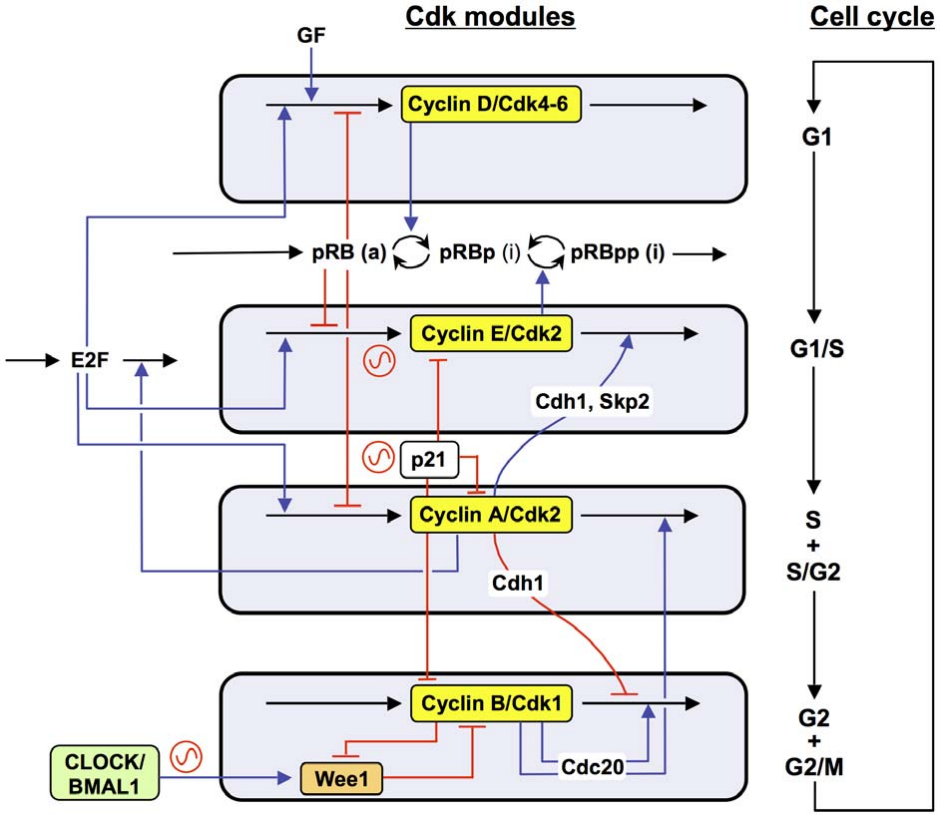
Scheme of the model for the Cdk network driving the mammalian cell cycle. The Cdk network contains four modules. Each of these modules is centered around one cyclin/Cdk complex: cyclin D/Cdk4–6 and cyclin E/Cdk2 promote progression in G1 and elicit the G1/S transition; cyclin A/Cdk2 ensures progression in S and the S/G2 transition, while cyclin B/Cdk1 brings about the G2/M transition. The growth factor (GF) promotes the G0/G1 transition by activating the synthesis of cyclin D. The model for the Cdk network also incorporates regulation by the Cdk inhibitor p21 (or p27) as well as the antagonistic effects of the transcription factor E2F and the tumor suppressor pRB; E2F promotes cell cycle progression by activating the synthesis of cyclins, which is repressed by pRB (see Ref. 14 for further details). The cell cycle network is coupled to the circadian clock through circadian variations in the levels of Wee1, p21 and cyclin E. The level of Wee1 is controlled by the circadian variation of the complex CLOCK-BMAL1, generated by the circadian clock; this complex also controls the levels of cyclin E (via c-Myc) and of REV-ERBα, which in turn controls the level of p21.

The model for the Cdk network presented in more detail in Ref. [14] is centered on the four main cyclin/Cdk complexes that control the orderly progression along the different phases of the mammalian cell cycle (Fig. 1). It contains 39 variables: besides the four cyclin/Cdk complexes it incorporates the tumor suppressor pRB and the transcription factor E2F; pRB inhibits progression in the cell cycle through repressing the transcription of various cyclins, while E2F promotes cell cycle progression by inducing their synthesis (see Ref. 14 for more details). The presence of growth factor permits the synthesis of cyclin D and allows cells to enter in the G1 phase (see Fig. 1). The cyclin D/Cdk4-6 complex phosphorylates and thereby inhibits the tumor suppressor pRB; this activates E2F and elicits the synthesis of cyclin E during G1. The cyclin E/Cdk2 complex further inhibits pRB through additional phosphorylation, which results in the full activation of E2F. The increase in cyclin E/Cdk2 elicits the G1/S transition. The transcription factor E2F also promotes the synthesis of cyclin A, which allows progression in the S phase. At the end of this phase the rise in cyclin A/Cdk2 brings about the S/G2 transition; by triggering the degradation of E2F and the subsequent decrease in synthesis of G1 cyclins it also brings an end to the previous phases of the cell cycle. Cyclin A/Cdk2 also promotes the accumulation of cyclin B through inhibition of Cdh1, which is involved in the degradation of cyclin B; the ensuing peak of activity of cyclin B/Cdk1 brings about the G2/M transition. In addition to regulations based on cyclin synthesis and degradation, the activity of Cdk1 and Cdk2 is inhibited through phosphorylation by the kinase Wee1, or activated through dephosphorylation by the phosphatases Cdc25. Negative and positive feedback loops abound in the network; the latter are exemplified by the dual action of cyclin B/Cdk1, which inhibits Wee1 and activates Cdc25. Finally, the various cyclin/Cdk complexes are regulated through the formation of inactive complexes with the Cdk inhibitor p21.

In our previous study [14] we showed that in the absence of growth factor (GF), the Cdk network evolves to a stable steady state characterized by low levels of cyclin/Cdk complexes, which corresponds to the quiescent, G0 phase of the cell cycle. Above a critical level of growth factor, the Cdk network undergoes sustained oscillations. Because each Cdk module activates the next module and inhibits the previous one in the network, these oscillations correspond to the repetitive, transient, ordered activation of the various cyclin/Cdk complexes, which brings about the successive phases of the cell cycle. The qualitative behavior of the model compares with the dynamics of the mammalian cell cycle in regard to (i) existence of a restriction point in G1 beyond which removal of GF does not prevent the cell to complete a cycle of Cdk2 and Cdk1 activation; (ii) balance between the antagonistic effects of pRB and E2F; (iii) repetitive cycling without need for GF in the absence of pRB; (iv) endoreplication, due to cyclic activation of Cdk2 without activation of Cdk1 [14]. Here we focus on another property of the model, which pertains to its entrainment by the circadian clock. We first consider the case where the cell cycle is coupled to the circadian clock via the kinase Wee1. This mode of coupling through a Cdk inhibitor was the first to be uncovered experimentally [10]. The model allows us to test how a cell cycle characterized by a period smaller or longer than 24 h can be entrained by the circadian variation of this inhibitor. We turn thereafter to the cases where coupling occurs via the Cdk inhibitor p21 or via cyclin E, before combining these multiple modes of coupling.

### Entrainment to 24 h or 48 h via the kinase Wee1

One mode of coupling between the cell cycle and the circadian clock is through the kinase Wee1 that inhibits Cdk1 and, thereby, the G2/M transition [10]. To determine its effect on the dynamics of the cell cycle, we must incorporate the circadian variation of the kinase Wee1. To this end we resort to a model proposed for the mammalian circadian clock [21] in which the complex CLOCK-BMAL1 oscillates with a circadian period. Coupling this model to the model for the Cdk network, we assume that the complex CLOCK-BMAL1 controls the transcription of the Wee1 gene (see Methods). In the original model for the mammalian cell cycle, we only considered the Wee1 protein, but not explicitly its mRNA, and assumed that its rate of synthesis remained constant.

**Figure 2:**
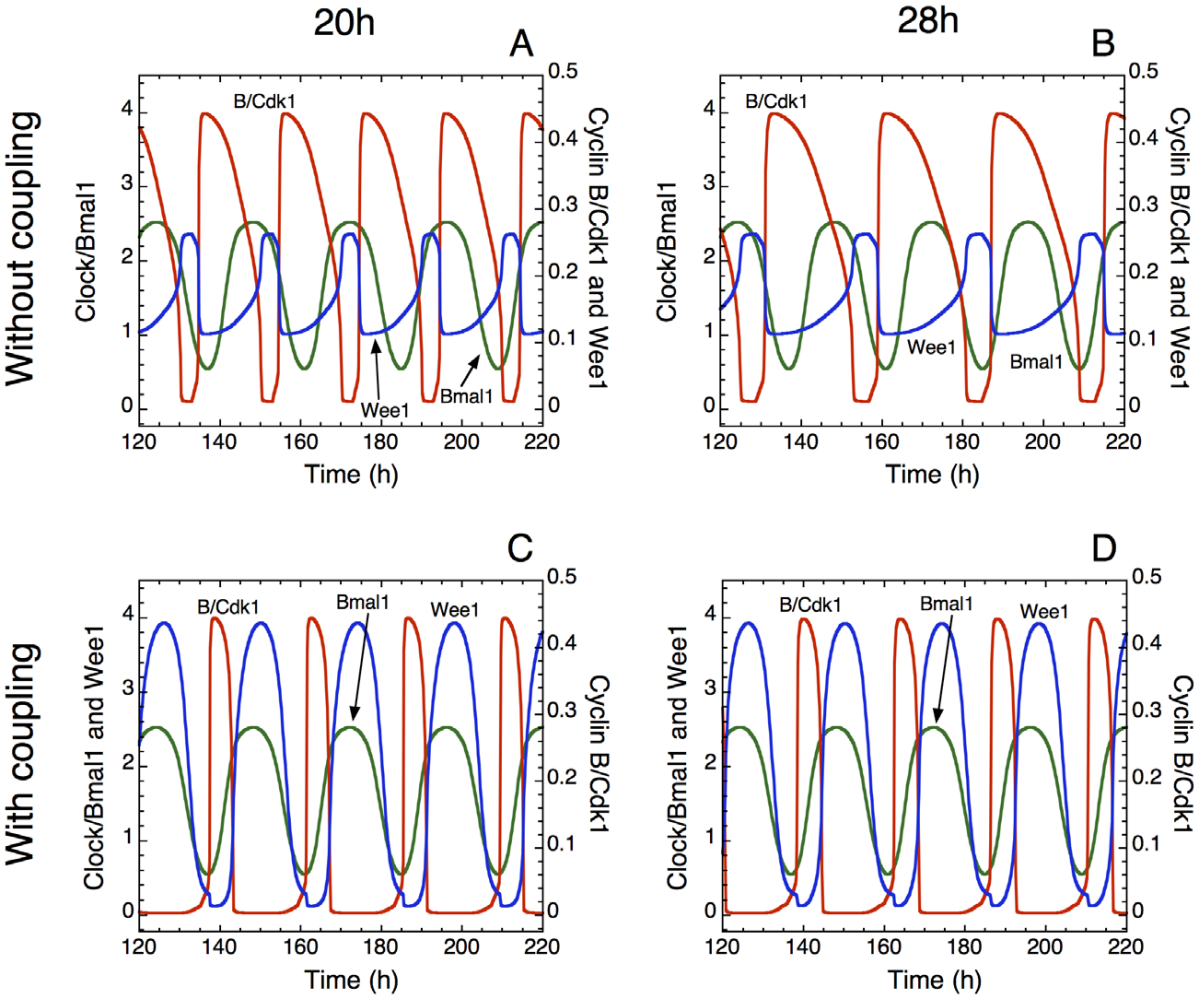
Entrainment of the cell cycle by the circadian clock through the kinase Wee1. The time series show the evolution of cyclin B/Cdk1 (red), the kinase Wee1 (blue), and the circadian complex CLOCK-BMAL1 (green) in the absence (A, B) or presence (C, D) of coupling to the circadian clock. The autonomous period of the cell cycle is either smaller (left column) or larger (right column) than 24 h; prior to coupling (upper row), the oscillations in the cell cycle (reflected by Wee1 and cyclin B/Cdk1) have then a period distinct from that of the circadian clock (reflected by CLOCK-BMAL1). In both cases the coupling to the circadian clock via Wee1 (see Methods) results in the entrainment of the cell cycle (lower row): the period then shifts from 20 h (A) to 24 h (C), and from 28 h (B) to 24 h (D), so that now Wee1, cyclin B/Cdk1 and CLOCK-BMAL1 all oscillate with a fixed phase relationship and with the same period equal to 24 h. The curves have been obtained by numerical integration of the kinetic equations [1]–[39] listed in the Supporting Information in Ref. 14, supplemented with eqs. [1]–[2] given in Methods. The latter equations include the variable CLOCK-BMAL1 whose time evolution is obtained by the concomitant numerical integration of eqs. [1]–[19] that govern the dynamics of the model for the mammalian circadian clock [21]; these equations are listed together with parameter values in the Supporting Information in Ref. 21 (see Methods). Parameter values for the Cdk network are those listed in Table S2 of [14], with *v*_cb_ = 0.055 µM h^−1^. Moreover, *v*_sw_ = 0 for A and B, and 0.1 µM h^−1^ for C and D; *K*_aw_ = 2 nM, *V*_dmw_ = 0.5 µM h^−1^, *K*_dmw_ = 0.5 µM, *nmw* = 4, and *k*_sw_ = 5 h^−1^.

Figure 2 shows the temporal evolution of CLOCK-BMAL1, Wee1, and cyclin B/Cdk1 in the absence (A, B) or in the presence (C, D) of coupling to the circadian clock through the kinase Wee1. Before coupling, the autonomous period of the cell cycle, characterizing the oscillations in Wee1 and cyclin B/Cdk1, is equal to 20 h (A, C) or 28 h (B, D). Since the kinase Wee1 is an inhibitor of the cell cycle, we can anticipate that upon coupling the cell cycle to the circadian clock via Wee1, progression in the cycle will be slowed down so that cell cycles shorter than 24 h may readily acquire a circadian period. This is shown in Fig. 2C where the period of the cell cycle increases from 20 h to 24 h upon coupling: this behavior represents entrainment of the cell cycle by the circadian clock. The question arises as to whether a coupling of the cell cycle to the circadian clock through the kinase Wee1 is also capable of entraining cell cycles longer than 24 h. Indeed, how could an inhibitor of cell cycle progression accelerate the cell cycle to entrain it to 24 h? As shown by the numerical simulations in Fig. 2D, such an entrainment can occur because the coupling through Wee1 reduces the width of the activity peaks of the various cyclin/Cdk complexes, and thereby accelerates progression in the cell cycle.

Some experimental evidence indicates that the inhibition of Cdk1 by phosphorylation prior to its degradation by APC at the end of mitosis contributes to exit from mitosis [22]. The model suggests that circadian activation of the synthesis of Wee1 augments the level of the protein, and thereby shortens the peak of activity of Cdk1 (see Fig. 2); according to the experiments, such circadian enhancement in Wee1 expression could shorten the duration of mitosis.

**Figure 3 pcbi-1002516-g003:**
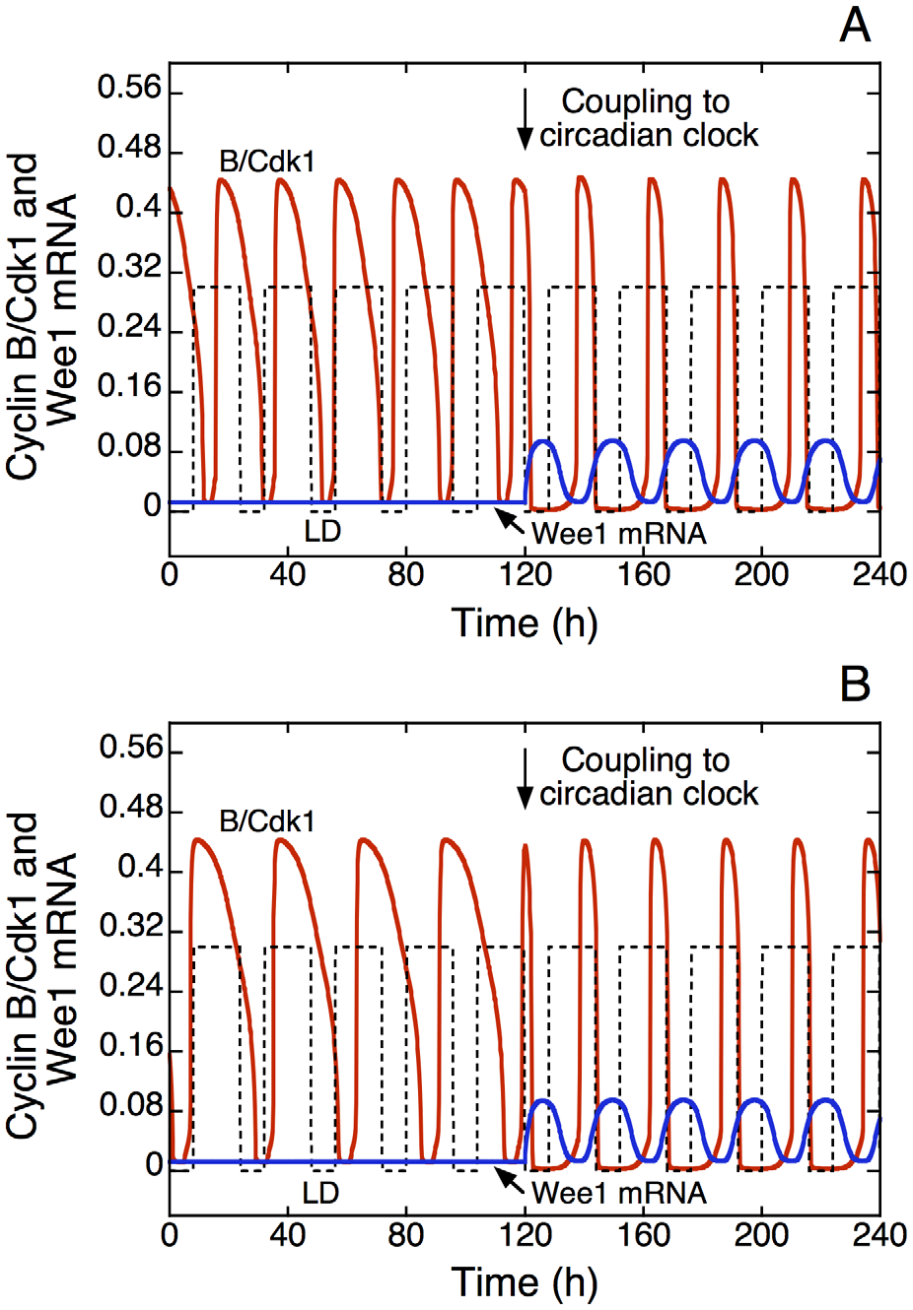
Phase of the cell cycle in the light-dark (LD) cycle upon entrainment by the circadian clock through the kinase Wee1. The time series show the time evolution of cyclin B/Cdk1 and of Wee1 mRNA in the absence (*v*_sw_ = 0 for t<120 h) or presence (*v*_sw_ = 0.1 µM h^−1^ for t>120 h) of coupling to the circadian clock, when the autonomous period of the cell cycle, prior to coupling via Wee1, is smaller (20 h, A) or larger (28 h, B) than 24 h. In both cases, cyclin B/Cdk1 peaks at the end of the L phase upon entrainment, in agreement with experimental observations [23]. The square wave (dashed line) represents the 24-h LD cycle; it corresponds to the increase above a basal value in the rate of synthesis, *v*_sP_, of *Per* mRNA during the L phase. This increment goes from zero in the D phase to 0.3 nM h^−1^ in the L phase. In all figures the durations of the L and D phases are equal to 16 h and 8 h, respectively. The values of the parameters in the model for the circadian clock are those corresponding to Fig. 8 of the Supporting Information in [21], with *K*_ib_ = 1 nM. The desired autonomous period of the cell cycle is achieved by adjusting the scaling parameter *eps* (see [14] and Methods), with *eps* = 21.58 in A, and 15.37 in B. Parameter values are the same as in Fig. 2.

When the cell cycle is entrained by the circadian clock, the phase angle between the two oscillatory processes remains constant. The circadian clock is itself entrained by the light-dark (LD) cycle through the periodic change in the rate of expression of the *Per* gene, which increases in the light phase. Therefore, upon entrainment by the circadian clock, the phase angle between the cell cycle and the LD cycle is fixed. Together with the LD cycle the time evolution of cyclin B/Cdk1 and of Wee1 mRNA is represented in Fig. 3 in the absence (t<120 h) or presence (t>120 h) of cell cycle entrainment by the circadian clock through Wee1, for an autonomous period of the cell cycle of 20 h (A) or 28 h (B). In both cases, upon entrainment the peak of cyclin B/Cdk1 occurs at the end of the light phase. This result fits with experimental observations showing that the circadian variation of cyclin B in oral mucosa peaks at the end of the light phase [23].

**Figure 4 pcbi-1002516-g004:**
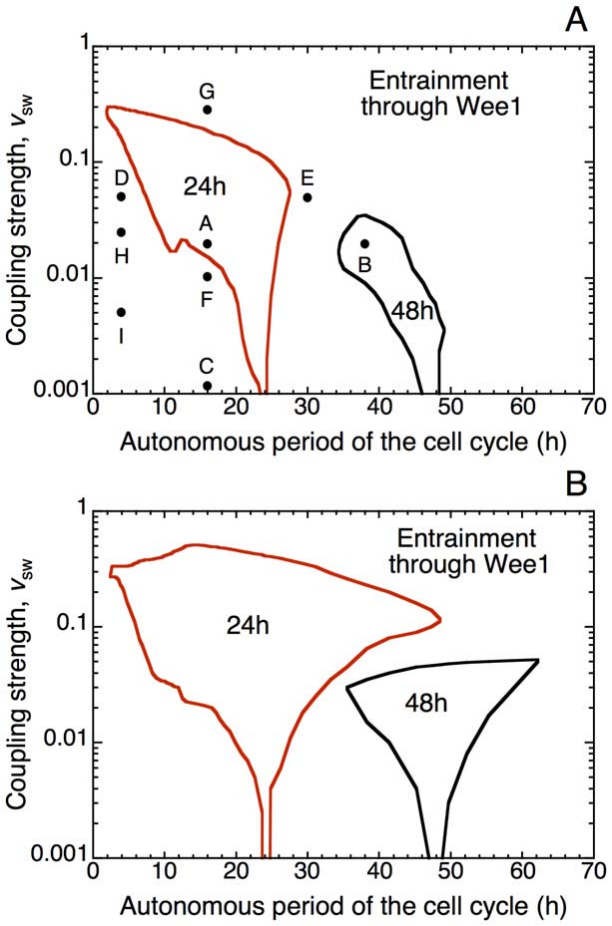
Domains of entrainment of the cell cycle by the circadian clock via circadian control of the kinase Wee1. The domains are determined as a function of the autonomous period of the cell cycle prior to coupling and of the strength of coupling (see text and Methods). We consider that the cell cycle is entrained when Cdk2 and Cdk1 both exhibit one large-amplitude peak per 24 h or 48 h. Entrainment of the cell cycle to 24 h occurs for autonomous periods smaller or larger than 24 h. The domains of entrainment are determined in the presence (A) or absence (B) of a basal rate of synthesis, *v*_swee1_, of the kinase Wee1, which is equal to 0.06 µM h^−1^ or 0, respectively. The size of the domains of entrainment of the cell cycle by the circadian clock increases as the basal rate of synthesis of Wee1 diminishes. Points A–I refer to situations illustrated in Figs. 5–7. The results are obtained as described in the legend to Fig. 2, for *v*_cb_ = 0.05 µM h^−1^. Other parameters values are the same as in Fig. 2.

We determined in a systematic manner the domain of entrainment of the cell cycle by the circadian clock via Wee1 as a function of the autonomous period of the cell cycle (i.e., its period before entrainment, in the absence of coupling) and of the strength of coupling, measured by the value of parameter *v*_sw_, which denotes the rate of Wee1 mRNA synthesis controlled by CLOCK-BMAL1. We take into account the possibility that the Wee1 protein is synthesized at a basal rate *v*_swee1_ independent of the circadian clock. Figure 4 illustrates the domain of entrainment to 24 h of the cell cycle by the circadian clock, through the kinase Wee1, in the presence (A) or absence (B) of a basal rate of synthesis of Wee1. The model shows that the domain of entrainment is more extended in the latter case. Then, periods of the cell cycle smaller or larger than 24 h are well entrained by the circadian clock: the range is quite extended since cell cycles going from slightly less than 5 h to almost 50 h can be entrained to 24 h. When the basal rate of Wee1 synthesis is not nil, as is likely the case in physiological conditions, the domain of entrainment decreases. Due to the cumulated effects of a basal and a circadian-clock controlled synthesis of the protein, Wee1 can reach higher values. If the level of Wee1 becomes too large, oscillations of large amplitude in Cdk1 are prevented and entrainment is ruled out.

**Figure 5 pcbi-1002516-g005:**
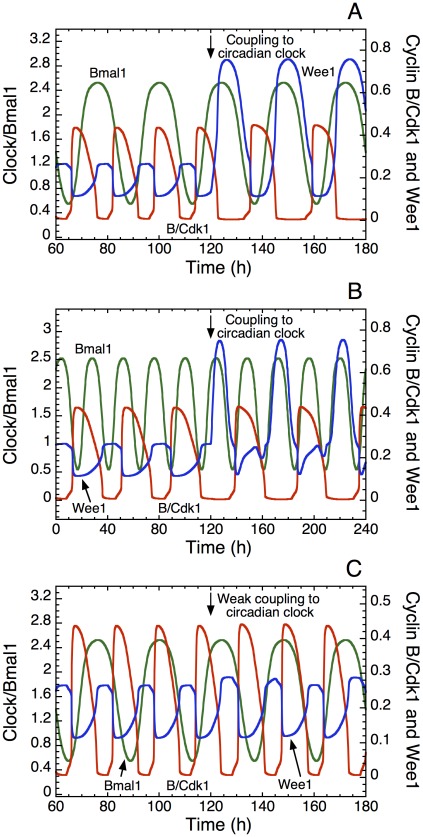
Entrainment of the cell cycle to 24 h or 48 h. The cell cycle is coupled to the circadian clock through the kinase Wee1. Shown in each panel are the time series for cyclin B/Cdk1 (red), the kinase Wee1 (blue) and the circadian complex CLOCK-BMAL1 (green) before (t<120 h) or after (t>120 h) coupling to the circadian clock. Conditions A–C correspond, respectively, to points A–C in Fig. 4A. (A) The cell cycle has an autonomous period of 16 h and is entrained to 24 h when the coupling strength, measured by parameter *v*_sw_ is sufficiently large. In t = 120 h, *v*_sw_ is raised from 0 to 0.02 µM h^−1^. (B) For the same coupling strength, i.e. for the same increase in *v*_sw_, entrainment to 48 h occurs when the autonomous period prior to coupling is 38 h. One peak in Cdk1 occurs for every second peak in BMAL1. (C) When the autonomous period is 16 h as in (A), the cell cycle fails to be entrained by the circadian clock when the coupling strength is reduced; here *v*_sw_ increases from 0 to 0.0012 µM h^−1^ in t = 120 h. Other parameters values are the same as in Fig. 4A.

**Figure 6 pcbi-1002516-g006:**
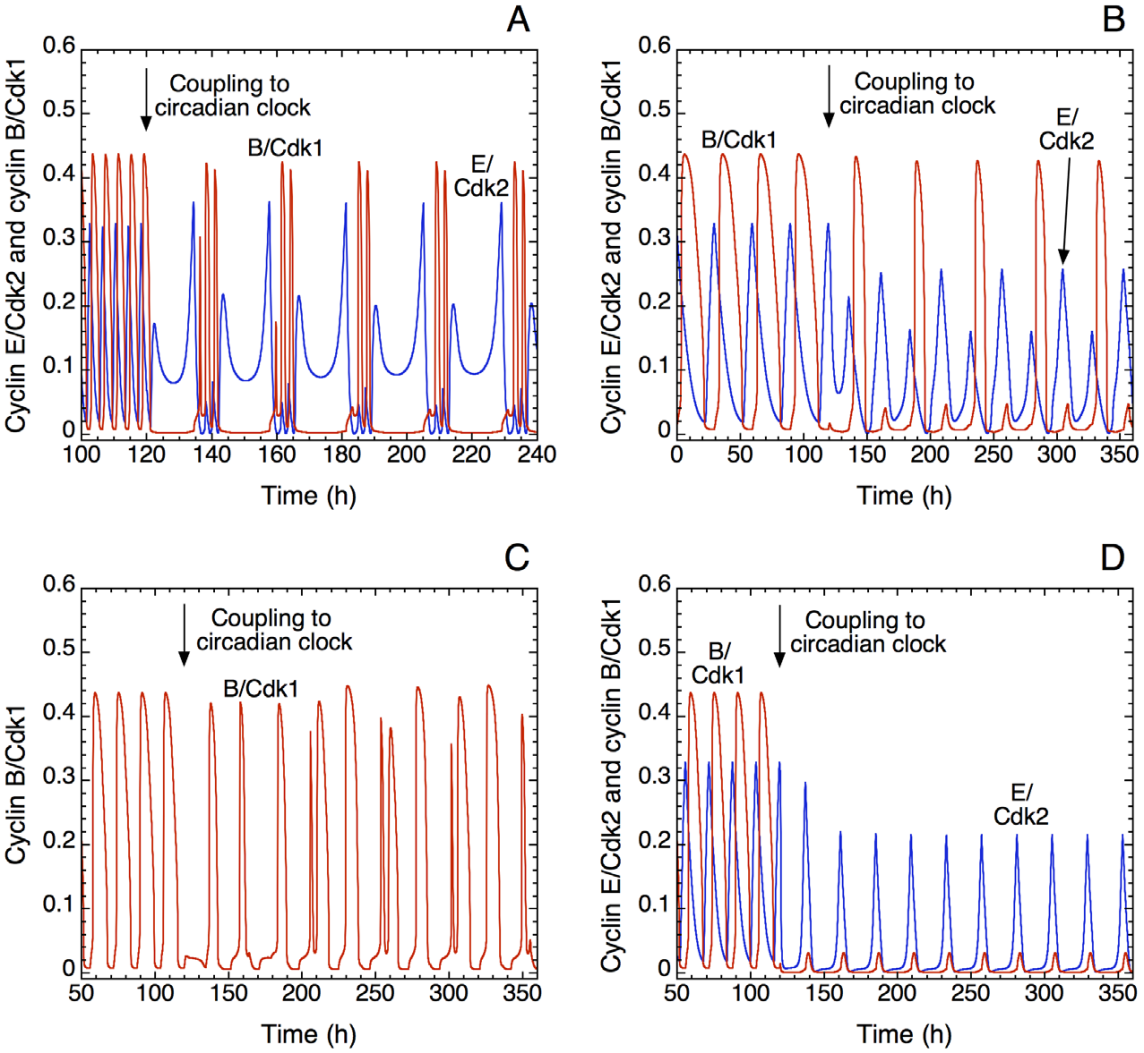
Complex oscillatory behavior of the cell cycle induced by coupling to the circadian clock. As in Figs. 2–5 and 7, coupling is achieved through the kinase Wee1. Shown is the time evolution of cyclin E/Cdk2 (blue) and cyclin B/Cdk1 (red) in the absence (t<120 h) or presence (t>120 h) of coupling to the circadian clock. Conditions A–D correspond, respectively, to the points D–G surrounding the domain of entrainment to 24 h in Fig. 4A. (A) Complex periodic oscillations. The autonomous period of the cell cycle is equal to 4 h and the coupling strength, measured by the rate constant for the synthesis of Wee1 mRNA, *v*_sw_, is equal to 0.05 µM h^−1^; the period of the cell cycle is too short to be well entrained by the circadian clock, and two peaks of cyclin B/Cdk1 appear every 24 h, together with a small and a large peak in cyclin E/Cdk2. (B) The autonomous period of the cell cycle is equal to 30 h and *v*_sw_ is still equal to 0.05 µM h^−1^. In this case, two peaks of cyclin E/Cdk2 appear for each peak of cyclin B/Cdk1. Such complex periodic oscillations would correspond to tetraploidy. (C) The autonomous period of the cell cycle is equal to 16 h and *v*_sw_ is equal to 0.01 µM h^−1^. Coupling is not strong enough to allow correct entrainment of the cell cycle by the circadian clock, resulting in irregular, chaotic oscillatory behavior (cyclin E/Cdk2 is not shown, for the sake of clarity). The chaotic nature of the oscillations was characterized by means of Poincaré sections, as described in Fig. 9 of [25]. (D) The autonomous period of the cell cycle is equal to 16 h and *v*_sw_ is equal to 0.3 µM h^−1^. Here, the coupling is so strong that it induces a high level of Wee1, which continuously inhibits the kinase Cdk1 while oscillations of Cdk2 persist. This simple periodic behavior would correspond to endoreplication. Other parameter values are as in Fig. 4A.

**Figure 7 pcbi-1002516-g007:**
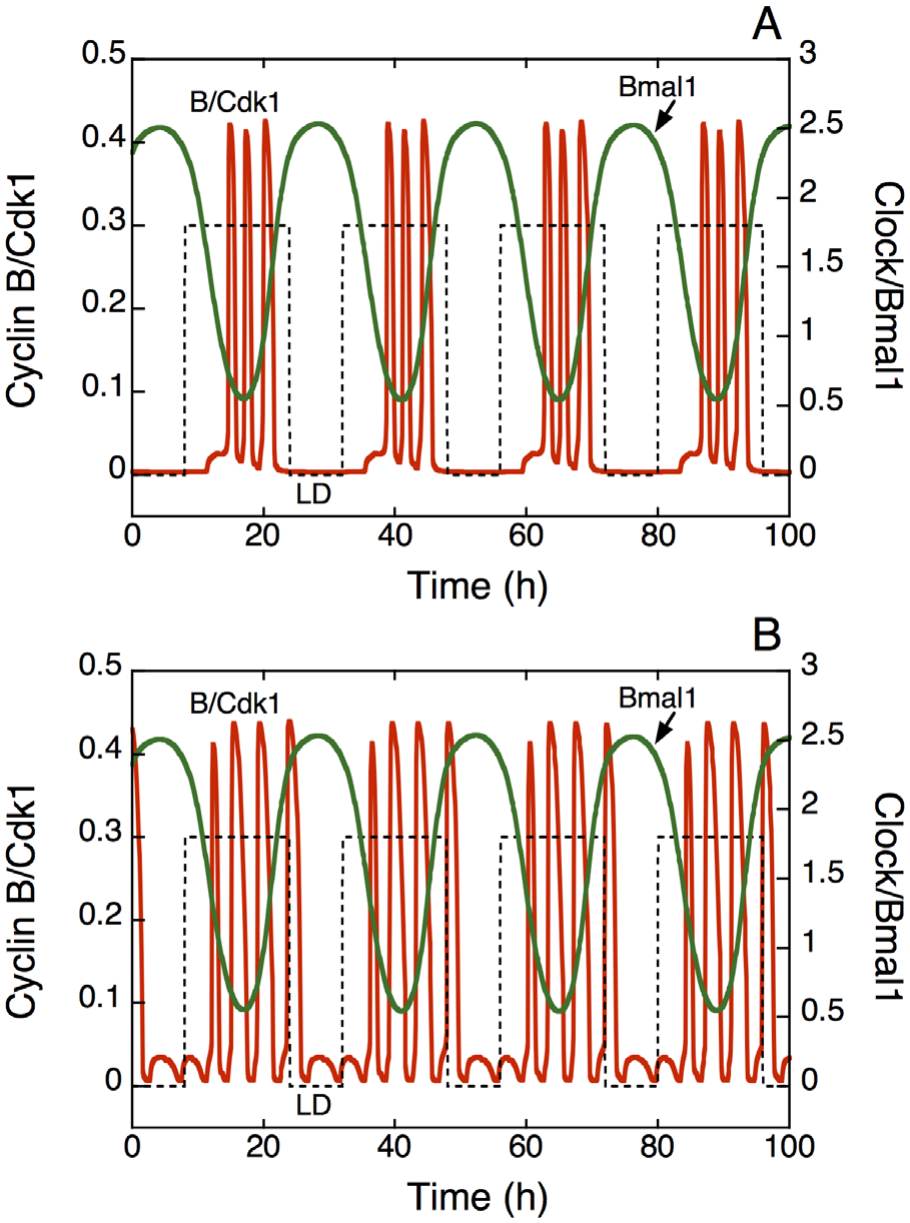
Circadian inhibition of rapid cell cycles due to coupling through Wee1. The curves show the time course of cyclin B/Cdk1 (red), CLOCK-BMAL1 (green), and the LD cycle (dashed line) when the autonomous period of the cell cycle prior to coupling is equal to 4 h, as in Fig. 6A, the coupling strength, *v*_sw_, is equal to 0.025 µM h^−1^ (A) or 0.005 µM h^−1^ (B). Panels (A) and (B) correspond, respectively, to points H and I in Fig. 4A. Whereas two peaks in cyclin B/Cdk1 occur in Fig. 6A at a higher coupling strength for the same autonomous period corresponding to point D in Fig. 4A, three or four peaks of cyclin B/Cdk1 occur during the L phase of the LD cycle when the coupling strength is progressively decreased. This is due to the accompanying decrease in the peak of the Cdk inhibitor Wee1 associated with the circadian peak in CLOCK-BMAL1 during the L phase. Other parameters values are the same as in Fig. 4A.

The domain of entrainment in Fig. 4 increases with the strength of coupling, then decreases until entrainment disappears at higher values of *v*_sw_ corresponding to large levels of the inhibitor Wee1. Because Wee1 inhibits Cdk1 more than Cdk2, the latter kinase can still oscillate and be entrained at a circadian period even in the absence of large amplitude oscillations in Cdk1, as will be shown further below.

At larger values of the autonomous period of the cell cycle, entrainment to double the circadian period, i.e. 48 h, can occur (Fig. 4A and B). Here also the domain of entrainment to 48 h is smaller in the presence of a basal rate of synthesis of Wee1 independent of the circadian clock. The domain of entrainment to 48 h is in each case reduced as compared to the domain of entrainment to 24 h. When the autonomous cell cycle period is close to 24 h or 48 h, we observe domains of entrainment, called *Arnold tongues*, which become narrower as the coupling strength decreases.

Cell cycle entrainment to 48 h is illustrated in Fig. 5B, which corresponds to point B in Fig. 4A. It shows the time evolution of cyclin B/Cdk1 before (t<120 h) or upon (t>120 h) entrainment by the circadian clock through the kinase Wee1. As soon as coupling is established in t = 120 h, the period of the cell cycle passes from 38 h to 48 h: a peak in cyclin B/Cdk1 is observed every second peak in BMAL1. Entrainment to 24 h is shown in Fig. 5A, which corresponds to point A in Fig. 4A: the coupling strength is the same as in Fig. 5B but the autonomous period here is equal to 16 h. If the coupling strength is not nil (as in Fig. 2) but too weak, entrainment fails to occur, as shown in Fig. 5C, which corresponds to point C in Fig. 4A: the autonomous period here also equals 16 h, but the coupling strength, measured by the maximum rate *v*_sw_ of Wee1 mRNA synthesis controlled by the circadian clock, is reduced by a factor of 20 as compared to the case in Fig. 5A.

What happens when the cell cycle is coupled to the circadian clock but fails to be entrained by it? We illustrate the different modes of dynamic behavior which can then be encountered by considering four points, marked D-G, surrounding the domain of entrainment to 24 h in Fig. 4A. The effect of the coupling on the time evolution of cyclin B/Cdk1 and cyclin E/Cdk2 in these points is shown in Fig. 6 A–D, respectively. In (A), the autonomous period of the cell cycle is equal to 4 h. Complex periodic oscillations occur as a result of coupling the cell cycle to the circadian clock: two peaks of cyclin B/Cdk1 and cyclin E/Cdk2 appear per 24 h (Fig. 6A for t>120 h), with a large interval separating the groups of twin peaks in Cdk1. The autonomous period is too small to allow entrainment with one peak of cyclin B/Cdk1 per 24 h. In (B), the autonomous period of the cell cycle is equal to 30 h. In the presence of coupling to the circadian clock (for t>120 h), per 24 h two peaks of cyclin E/Cdk2 appear for each peak of cyclin B/Cdk1. Such behavior could correspond to tetraploidy. In (C), the autonomous period of the cell cycle is equal to 16 h but the strength of the coupling to the circadian clock is not large enough to entrain the cell cycle to 24 h: here, in contrast to Fig. 5A where entrainment occurs for the same autonomous period of the cell cycle but with stronger coupling to the circadian clock, the coupling via Wee1 results in complex, irregular oscillations corresponding to chaotic behavior. Finally, in (D), the autonomous period of the cell cycle is also equal to 16 h, but the strength of the coupling to the circadian clock is too large for entrainment to occur. Let us recall that we define circadian entrainment of the cell cycle as the occurrence of one large-amplitude peak in both Cdk1 and Cdk2 with a periodicity of 24 h or 48 h; deciding whether the peak in Cdk1 has a large amplitude or not is made easy by the presence of bistability in the Cdk1 module, because of which the amplitude of the peak of cyclin B/Cdk1 in the course of oscillations is either minute or very large [14]. In Fig. 6D, the elevated level of kinase Wee1 causes the continuous inhibition of cyclin B/Cdk1 and, to a lesser degree, of cyclin A/Cdk2 and cyclin E/Cdk2. In this case, the model predicts that only the kinase Cdk2 will be entrained to oscillate at 24 h with large amplitude, whereas Cdk1 undergoes sustained oscillations of only minute amplitude. This behavior corresponds to repetitive cycles of DNA replication without mitosis, a phenomenon known as endoreplication [24]. Endoreplication can also occur in the model in autonomous conditions, i.e. in the absence of coupling to the circadian clock [14], [25]. Altogether the diagram in Fig. 4A indicates that over a sizeable range of autonomous period of the cell cycle, entrainment to 24 h, and also 48 h, can occur in a range of intermediate strength of coupling between the cell cycle and the circadian clock.

The results presented above indicate that the coupling of the cell cycle to the circadian clock can give rise to various modes of oscillatory behavior, from entrainment to entrainment failure accompanied by complex periodic or aperiodic oscillations. It is probable that not all of these modes of dynamical behavior are physiologically relevant, and some are likely to occur more frequently than others *in vivo*. It is nevertheless useful to bring to light the full repertoire of possible dynamic behavior, since some of the more exotic modes of oscillations could well result from alterations of the coupling between the two cellular rhythms. The prediction that endoreplication and/or tetraploidy may occur if the coupling strength between the cell cycle and the circadian clock is too high (see Fig. 4A and Figs. 6B and D), could be tested experimentally by enhancing the circadian expression of the kinase Wee1.

That coupling to the circadian clock might sometimes lead to highly complex or even chaotic oscillations in the cell cycle might be less relevant from a physiological point of view than entrainment, endoreplication or tetraploidy. Complex periodic or aperiodic behavior could indeed be more difficult to observe experimentally because cells will likely induce molecular mechanisms leading to apoptosis if they enter into such irregular dynamical regimes.

In determining the effects of coupling the cell cycle to the circadian clock, we explored in Figs. 4, 6 and 7 a large range of durations of the cell cycle, from very small (a few hours) to very large (up to 60 h), so as to determine the full boundaries of the domains of entrainment to 24 h or 48 h. This led us to also consider periods of the cell cycle much shorter than 24 h, even if such small periods are likely to be rare. Indeed, periods of the cell cycle in mammals are usually larger than 10 h. However, periods as short as 4 h have been observed during rat gastrulation [26]. The results highlight the theoretical possibility of entraining such short periods by the circadian clock, even though such periods might not be the most commonly encountered in physiological conditions.

We have seen in Fig. 6A that more than one peak of Cdk1 can occur per 24 h. For a given value of the autonomous period, the number of peaks in Cdk1 increases as the coupling strength measured by parameter *v*_sw_ decreases. Thus, when the autonomous period of the cell cycle is equal to 4 h, the number of peaks in Cdk1 per 24 h passes from 2 (Fig. 6A corresponding to point D in Fig. 4A) to 3 (Fig. 7A, corresponding to point H in Fig. 4A), and then to 4 (Fig. 7B, corresponding to point I in Fig. 4A) as the coupling strength is progressively decreased. This situation induced by coupling to the circadian clock would correspond to multiple rounds of mitosis over 24 h, a situation that has been observed experimentally [6]. The model predicts that the multiple peaks in Cdk1 all occur during the L phase. This is due to the phase of BMAL1, which peaks during the D phase of the LD cycle (Fig. 7). Indeed BMAL1 oscillates in antiphase with PER, which peaks at the end of the L phase since *Per* gene expression is triggered by light [27]. As a result, since BMAL1 induces the expression of the kinase Wee1 that inhibits Cdk1, the peaks in Cdk1 are prevented in the D phase and, consequently, peaks in Cdk1 (and mitosis) are gated by the circadian clock in the L phase. For the same reason, upon entrainment of the cell cycle by the circadian clock with a single peak of Cdk1 per 24 h, as in Fig. 3, the peak in Cdk1 occurs at the end of the L phase, in agreement with experimental observations [23].

### Entrainment via the Cdk inhibitor p21

Besides the well-known circadian regulation of the cell cycle through induction of the kinase Wee1 by the complex CLOCK-BMAL1 [10], others modes of coupling of the cell cycle to the circadian clock have been identified. The synthesis of the Cdk inhibitor p21 is transcriptionally controlled in a circadian manner: CLOCK-BMAL1 induces the expression of REV-ERBα, which in turn impedes the expression of p21 [11]. Whereas in the case of coupling through Wee1 the circadian clock inhibits progression in the cell cycle, here it has an opposite effect, since it decreases the level of a Cdk inhibitor.

**Figure 8 pcbi-1002516-g008:**
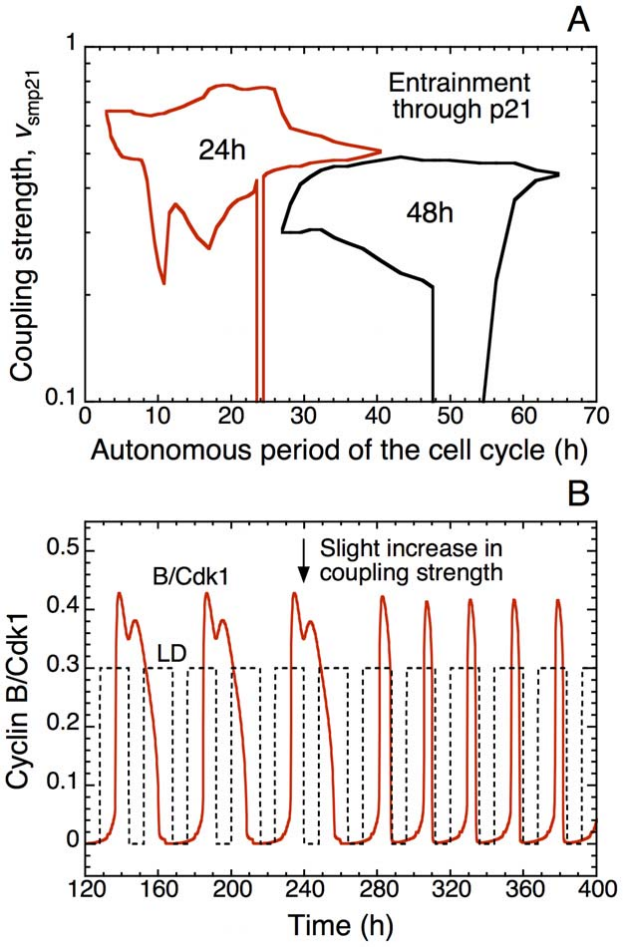
Entrainment of the cell cycle through the Cdk inhibitor p21 induced by the circadian clock protein REV-ERBα. (A) Domains of entrainment of the cell cycle by the circadian clock at 24 h and 48 h. As in Fig. 4, periods of the cell cycle smaller or larger than 24 h can be entrained at 24 h by the circadian clock, although the domain enlarges for periods less than 24 h. The two domains are close to each other when the autonomous period is in the range 30 h–40 h. (B) Time series showing the switch from entrainment of the cell cycle by the circadian clock at 48 h (t<240 h) to entrainment at 24 h (t>240 h) resulting from an increase of about 6.5% in coupling strength, when the autonomous period is equal to 34 h. The light-dark (LD) cycle is represented by a dashed line; the high and low portions of the square wave correspond to the L (16 h) and D (8 h) phases, respectively. In the presence of entrainment at 24 h, cyclin B/Cdk1 peaks at the end of the L phase, in agreement with experimental observations [23]. Parameter values in eqs. [3] and [4] (see Methods) are: *K*_ip21_ = 0.05 nM, *nmp21* = 1, *V*_dmp21_ = 0.5 µM h^−1^, *K*_dmp21_ = 0.5 µM, and *v*_s1p21_ = 50 h^−1^. Moreover, in (B), at t = 240 h parameter *v*_smp21_ measuring coupling strength increases from 0.46 µM h^−1^ to 0.49 µM h^−1^. As in Fig. 2, the values of the parameters in the model for the circadian clock are those corresponding to Fig. 8 of the Supporting Information in Ref. [21] with *K*_ib_ = 1 nM. While the shift in entrainment pattern in (B) follows from a permanent change in parameter value (here the coupling strength), a similar switch between the two modes of entrainment could also result from a transient perturbation, because in the region where the two domains of entrainment are close to each other in (A), numerical simulations indicate that the two stable modes of entrainment to 24 h and 48 h may sometimes coexist in the same conditions. This coexistence phenomenon corresponds to birhythmicity [1], [36].

The domain of entrainment of the cell cycle by the circadian clock through the protein p21 via REV-ERBα is shown in Fig. 8A as a function of the autonomous period of the cell cycle prior to coupling and of the coupling strength measured here by the maximum rate of p21 mRNA synthesis controlled (negatively) by REV-ERBα (see Methods). Similarly to entrainment through kinase Wee1, cell cycles with periods smaller or larger than 24 h can be entrained to oscillate at 24 h. Autonomous periods as large as 40 h can be reduced to 24 h upon coupling to the circadian clock via p21. For larger autonomous periods the cell cycle can be entrained to 48 h. As in Fig. 4, when the autonomous cell cycle period is close to 12 h, 24 h or 48 h, we observe Arnold tongues where entrainment occurs at smaller coupling strengths. When the autonomous period ranges between 32 h and 40 h in the case illustrated in Fig. 8A, we observe (see Fig. 8B) that the cell cycle coupled to the circadian clock can switch abruptly from a period of 48 h to a period of 24 h upon increasing the coupling strength only slightly, by less than 10%. This switch could also result from some transient perturbation, because the two modes of entrainment sometimes coexist in the same conditions, in the region where the two domains of entrainment are close to each other.

As shown in the right part of Fig. 8B, here also the peak of cyclin B/Cdk1 in the presence of circadian entrainment occurs during the second half of the light phase. Indeed, BMAL1 induces the expression of REV-ERBα, which leads, after a delay, to the rise of the protein REV-ERBα; the latter inhibits the expression of p21, leading to the decrease, after an additional delay, in the protein p21 that inhibits Cdk1. Although BMAL1 reaches a maximum in the D phase (see above and Fig. 7), the rise in REV-ERBα and the subsequent decrease in p21 will occur, because of the cumulated delays, in the second half of the L phase. The circadian clock therefore gates the peak in Cdk1 near the end of that phase.

### Entrainment via cyclin E

The circadian clock complex CLOCK-BMAL1 negatively regulates the expression of the oncogene c-Myc, which favors progression in the cell cycle by promoting the synthesis of the different cyclins, mainly in the G1 phase [12], [13]. For simplicity, we consider in the model that CLOCK-BMAL1 directly represses the synthesis of cyclin E only. The coupling here is expressed through the rate of synthesis of cyclin E mRNA, *v*_sce_, which is negatively controlled by the circadian clock.

**Figure 9 pcbi-1002516-g009:**
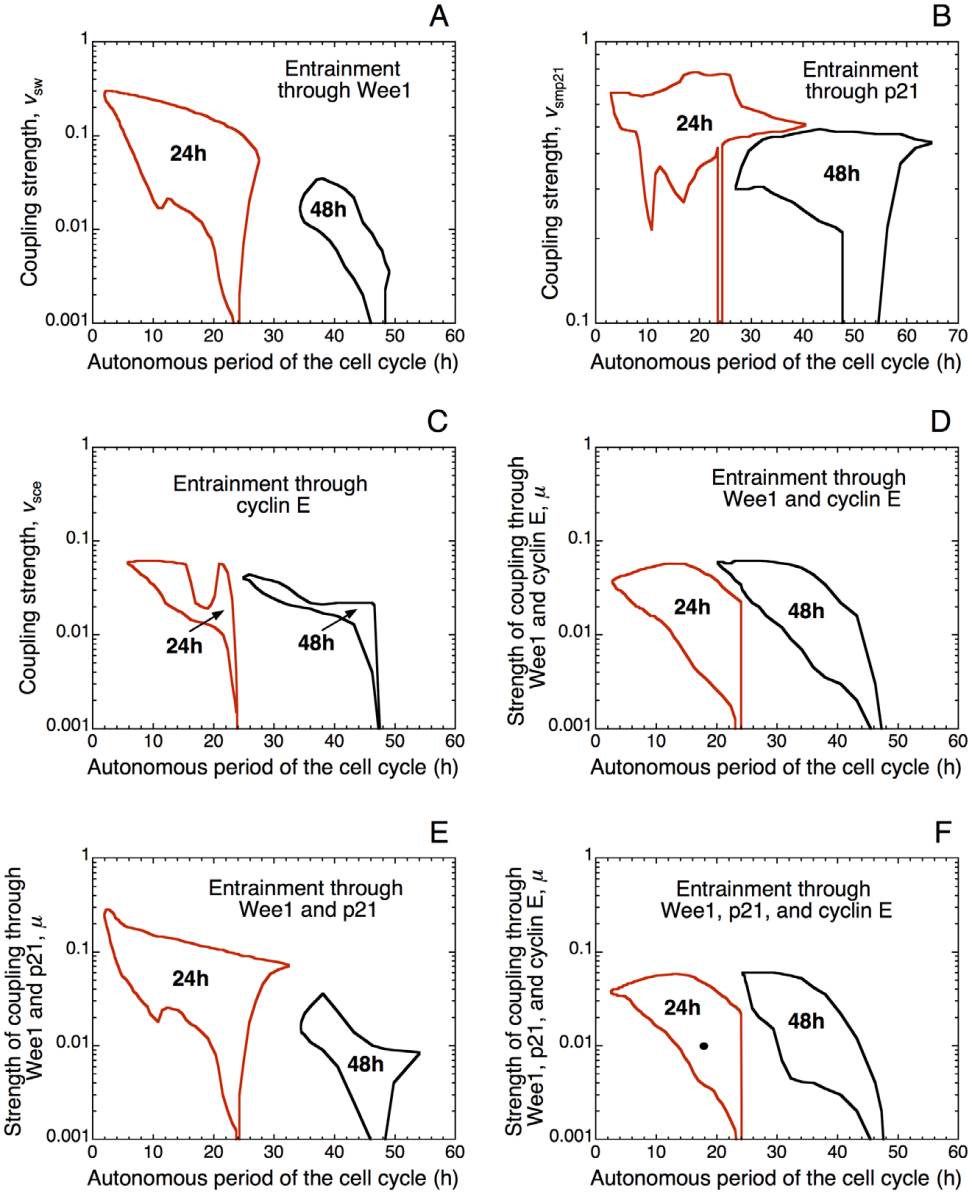
Entrainment of the cell cycle at 24 h or 48 h by the circadian clock through multiple modes of coupling. (A) Domains of entrainment through the kinase Wee1 in the presence of a basal rate of synthesis of the protein, *v*_swee1_ = 0.06 µM h^−1^. (B) Entrainment through the Cdk inhibitor p21. (C) Entrainment through the circadian regulation of the synthesis of cyclin E. (D) Entrainment of the cell cycle by the circadian clock through Wee1 and cyclin E. (E) Entrainment through Wee1 and p21. (F) Entrainment through Wee1, p21, and cyclin E. The black dot in (F) refers to the conditions in which entrainment through multiple modes of coupling occurs in Fig. 10. In (C), (D) and (F), parameter values in eqs. [5] and [6] (see Methods) are: *K*_ice_ = 1 nM, *V*_dmce_ = 0.5 µM h^−1^, *K*_dmce_ = 0.5 µM, *nce* = 4, *k*_ce2_ = 5 h^−1^. The strength of coupling through cyclin E is measured by the rate of synthesis of cyclin E mRNA depending on CLOCK-BMAL1, *v*_sce_. When multiple modes of coupling operate simultaneously, we multiply the rate of synthesis of *Mw*, *Mp21*, or *Mce* in eqs. [1]–[6] by the parameter μ, and put the value of *v*_sw_, *v*_smp21_, or *v*_sce_ equal to 1 µM h^−1^; μ then measures the strength of coupling to the circadian clock (see Methods where the various modes of coupling are detailed). The diagrams in (A) and (B) are the same as those of Fig. 4A and 8A, respectively; they are reproduced here to facilitate comparison with the other modes of entrainment.

The domain of entrainment of the cell cycle by the circadian clock through repression of cyclin E synthesis by CLOCK-BMAL1 is shown in Fig. 9C as a function of the coupling parameter *v*_sce_ and of the autonomous period of the cell cycle. For such a coupling, entrainment to 24 h is clearly favored when the autonomous period is less than 24 h. The same observation holds for the domain of entrainment at 48 h, which is observed only for autonomous periods shorter than 48 h. The size of the domains of entrainment appears to be reduced as compared to that observed for the coupling through Wee1 (compare Figs. 9C and 9A; the different scale in Fig. 9B established for the coupling through p21 makes the comparison less straightforward). One possible reason for the decrease in the size of the domain of entrainment is that cyclin E is more remote than Cdk1 from the core of the oscillatory mechanism in the Cdk network [14] and, as a consequence, it might be more difficult to entrain the Cdk network via cyclin E than via Wee1 or p21 which both act directly on Cdk1.

**Figure 10 pcbi-1002516-g010:**
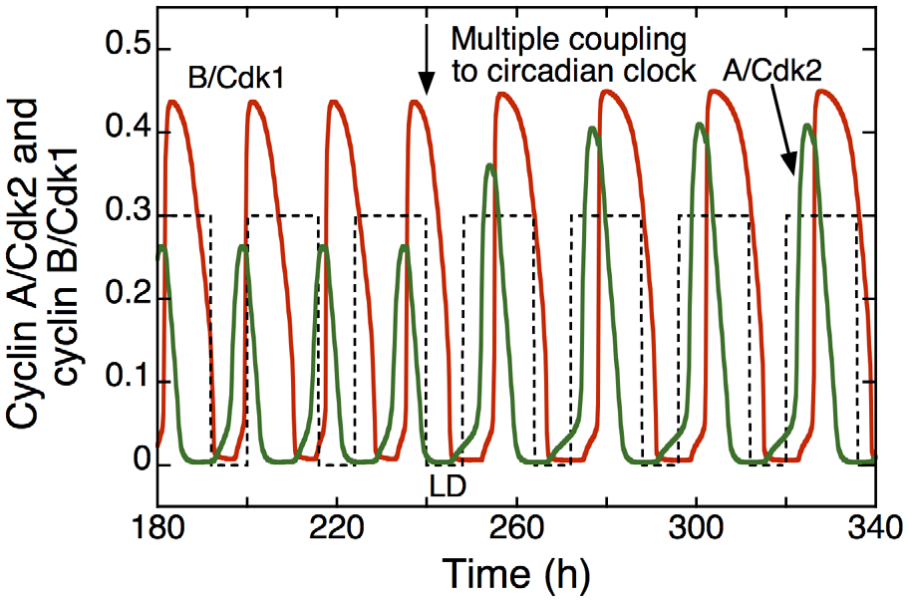
Entrainment of the cell cycle by the circadian clock in the presence of multiple modes of coupling through Wee1, p21, and cyclin E. Conditions correspond to the black dot in Fig. 9F: the cell cycle period changes from 18 h to 24 h upon coupling (vertical arrow). Shown is the time evolution of cyclin A/Cdk2 (green) and cyclin B/Cdk1 (red) in the absence (t<240 h) or presence (t>240 h) of multiple coupling to the circadian clock. The dashed line represents the LD cycle, as explained in the legend to Figs. 3 and 8. Parameter μ, which measures the coupling strength (see Methods), is equal to 0 for t<240 h and 0.01 for t>240 h.

### Entrainment via multiple modes of coupling

So far we have determined the domains of entrainment of the cell cycle by the circadian clock under different conditions of coupling, via Wee1, p21 or cyclin E. What happens when several components of the cell cycle are simultaneously controlled by the circadian clock? Does multiple coupling facilitate entrainment? To address this issue, we compare the domains of entrainment observed for coupling through only Wee1 (Fig. 9A), p21 (Fig. 9B), or cyclin E (Fig. 9C) with the domains obtained when coupling occurs through both Wee1 and cyclin E (Fig. 9D) or Wee1 and p21 (Fig. 9E), and finally with the domain of entrainment when all three modes of coupling operate simultaneously (Fig. 9F). The somewhat surprising conclusion of this comparison is that the size of the domain of entrainment does not increase when two or more modes of coupling link the cell cycle to the circadian clock. Indeed, the domain of entrainment via the simultaneous coupling through Wee1, p21 and cyclin E in Fig. 9F is not larger than the domain of entrainment only through Wee1, p21 or cyclin E. Entrainment through two modes of coupling yields domains of comparable magnitude. As to the phase upon entrainment, the peak in Cdk1 still occurs in the second half of the L phase when coupling occurs through Wee1, p21 and cyclin E simultaneously (Fig. 10).

We might have expected a broader domain of entrainment through the combination of multiple modes of coupling. The model shows that this is not the case, which suggests that the multiplicity of the modes of coupling, rather than facilitating entrainment, allows for redundancy. The multiplicity of links ensures that the cell cycle can still be entrained when some of the various modes of coupling to the circadian clock are nonfunctional or suppressed.

The model predicts that the coupling between the cell cycle and the circadian clock through the kinase Wee1 ensures the largest domain of circadian entrainment of the cell cycle. This may be due to the fact that Wee1 mainly regulates the Cdk1 module, which is crucial for the control of mitosis. The Cdk inhibitor p21 and cyclin E exert upstream regulation in the Cdk network, and are not directly linked to the core oscillatory mechanism controlling mitosis, which rests on the Cdk1 module. Thus, the entrainment of the cell cycle by the circadian clock through these modes of coupling might be less efficient than the coupling through the kinase Wee1.

Even if the coupling through the kinase Wee1 represents, in the model, the most robust way to entrain the cell cycle by the circadian clock, the model predicts that without coupling through Wee1, the redundancy in coupling permits entrainment of the cell cycle by the circadian clock via the other modes of coupling, through p21 and cyclin E. The domain of entrainment through both cyclin E and p21 (result not shown) is very similar to the domain of entrainment through cyclin E alone. The coupling through cyclin E and p21 thus reflects an apparent lack of synergy since the simultaneous presence of these two modes of coupling, does not enlarge the entrainment of the cell cycle by the circadian clock. Ensuring redundancy in the entrainment of the cell cycle by the circadian clock might therefore be more important than enlarging the range of periods and of coupling strengths over which entrainment occurs.

### Effect of the DNA replication checkpoint

**Figure 11:**
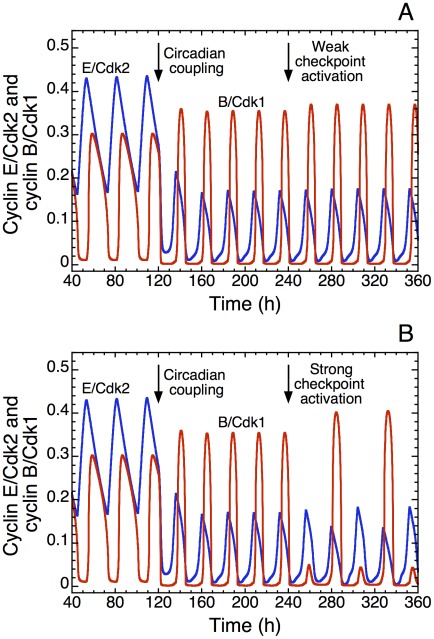
Entrainment by the circadian clock in the presence of a cell cycle checkpoint. The curves illustrate the effect of an activation of the ATR/Chk1 DNA replication checkpoint in the presence of coupling to the circadian clock through the kinase Wee1. Shown is the time evolution of cyclin E/Cdk2 (blue) and cyclin B/Cdk1 (red) in the absence (t<120 h) or presence (t>120 h) of coupling. For t>240 h, the DNA replication checkpoint is activated (second vertical arrow) by increasing the rate of synthesis of the kinase ATR, *k*_aatr_, which passes from 0 to 0.025 µM^−1^ h^−1^ (weak activation) in (A) or from 0 to 0.075 µM^−1^ h^−1^ (strong activation) in (B). The autonomous period of the cell cycle prior to coupling is equal to 28 h; similar results are obtained for the case where the cell cycle autonomous period is equal to 20 h. Numerical simulations show that weak activation of the checkpoint will not perturb circadian entrainment (A), while stronger activation leads to tetraploidy where two peaks of cyclin E/Cdk2 are present per peak of cyclin B/Cdk1 (B). In these simulations, the basal rate of synthesis of kinase Wee1, *v*_swee1_, is set equal to 0. Coupling to the circadian clock is achieved by raising the rate of synthesis of Wee1 mRNA, *v*_sw_, from 0 to 0.1 µM h^−1^ in t = 120 h (first vertical arrow). Other parameter values are as in Fig. 4.

We previously showed that incorporation of the ATR/Chk1 DNA replication checkpoint leads to a better separation of the peaks in the various cyclin/Cdk complexes without altering the oscillatory nature of the dynamics of the Cdk network in the presence of sufficient amounts of growth factor [14]. Here we wish to determine the effect of an activation of the ATR/Chk1 DNA replication checkpoint on the entrainment of the cell cycle by the circadian clock. Shown in Fig. 11 is the time evolution of cyclin E/Cdk2 and cyclin B/Cdk1 in the absence (t<120 h) or presence (t>120 h) of coupling to the circadian clock through Wee1. The autonomous period is equal to 28 h, and entrainment to 24 h is seen to occur upon coupling through Wee1 (first arrow). For t>240 h, the ATR/Chk1 DNA replication checkpoint is activated. The model shows that a low activation of the DNA replication checkpoint (second arrow in Fig. 11A) does not perturb entrainment of the cell cycle by the circadian clock. However, stronger activation of the DNA replication checkpoint (second arrow in Fig. 11B) can modify the dynamics of the cell cycle coupled to the circadian clock. Indeed, two peaks of cyclin E/Cdk2 may then occur for one peak of cyclin B/Cdk1. This behavior can be associated with tetraploidy. Experimentally, it was shown accordingly that hyperactivation of the DNA replication checkpoint can enhance the occurrence of tetraploid cells [28].

The model predicts that the effect of the DNA replication checkpoint on the dynamics of the cell cycle might become attenuated in the presence of strong coupling between the cell cycle and the circadian clock. Indeed, one effect of the DNA replication checkpoint is to slow down progression in the cell cycle to give enough time to the cell to progress correctly in the successive phases of the cell cycle and/or to repair cell damage. For autonomous periods of the cell cycle smaller than 24 h, the circadian clock will also slow down the progression in the cell cycle so as to entrain the cell cycle at a 24 h period. Thus, in the case of rapid cell cycles, the circadian clock and the DNA replication checkpoint both act to slow down progression in the cell cycle. However, for autonomous periods of the cell cycle larger than 24 h, the entrainment of the cell cycle by the circadian clock will tend to accelerate progression in the cell cycle. The question arises as to whether this might impede the operation of the DNA replication checkpoint. The model raises the possibility of an interference between these two processes.

### Circadian entrainment from a quiescent state

**Figure 12:**
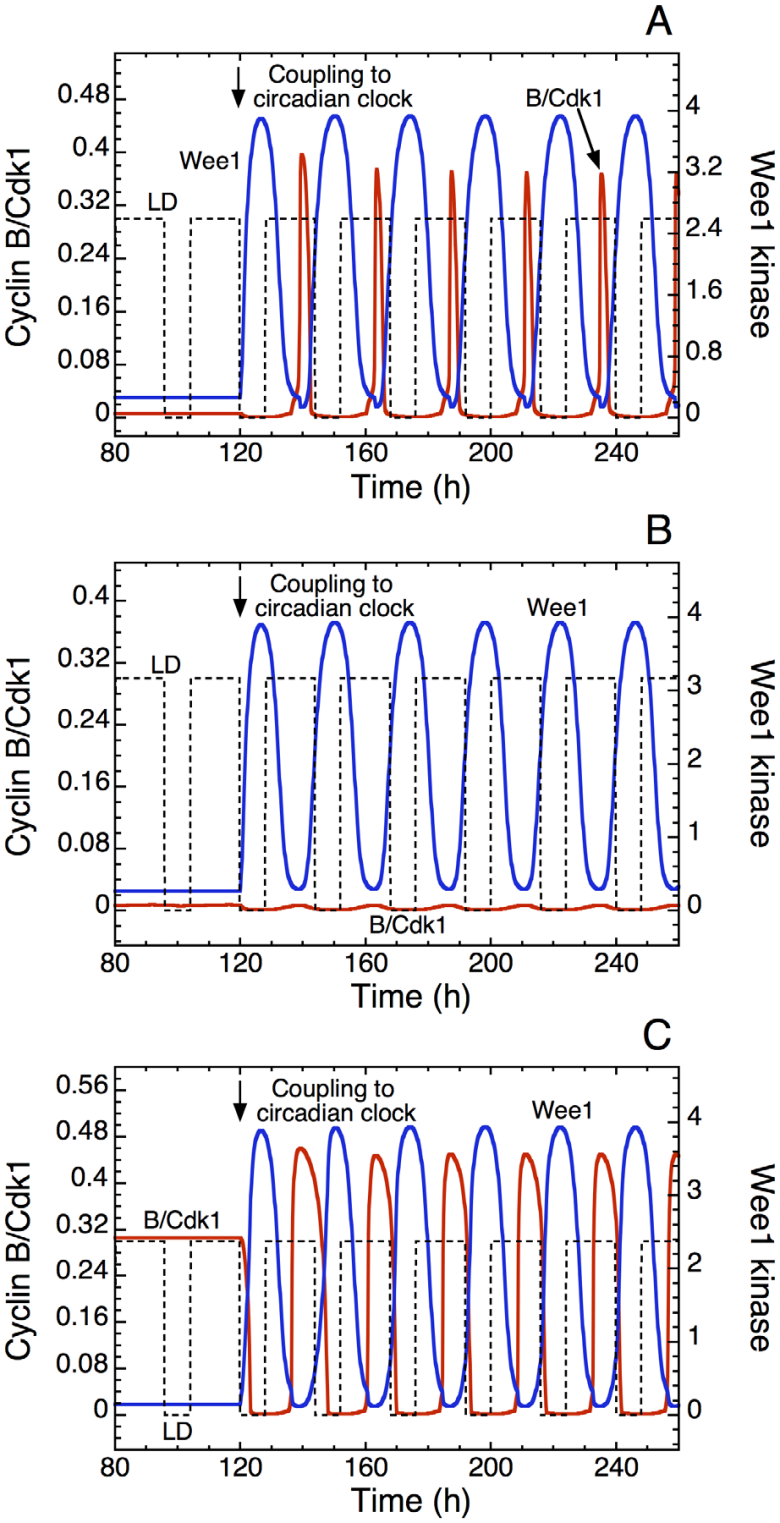
Circadian entrainment of a quiescent cell. (A) Upon raising the rate of pRB synthesis, *v*_spRB_, above a critical value, and in the absence of coupling to the circadian clock (0<t<120 h) the Cdk network reaches a stable steady state associated with quiescence [14]. Coupling to the circadian clock through Wee1 is achieved by raising the rate of synthesis of Wee1 mRNA, *v*_sw_, from 0 to 0.1 µM h^−1^ in t = 120 h (vertical arrow); this results in the entrainment of the Cdk network, reflected by the circadian variation of cyclin B/Cdk1 (red). (B) Further increase in the rate of synthesis of pRB still yields a stable steady state, but entrainment by the circadian clock fails to occur, as only tiny variations in the level of cyclin B/Cdk1 are observed. (C) Instead of increasing pRB, we increase the level of the phosphatase Cdc25 acting on Cdk1. The Cdk network then reaches a stable steady state characterized by a high level of cyclin B/Cdk1 [14]. Circadian entrainment of Cdk1 through the circadian variation in Wee1 (blue) is also obtained in such conditions. Parameter *v*_spRB_, which is equal to 0.8 µM h^−1^ in Figs. 2–11, is raised up to 1 µM h^−1^ in (A) and 2 µM h^−1^ in (B). In (C), the rate of synthesis of Cdc25, *v*_spbi_, which is equal to 0.12 µM h^−1^ in all other figures, is raised to 0.3 µM h^−1^, with *v*_spRB_ = 0.8 µM h^−1^. Other parameters are in Fig. 4A.

Entrainment by the circadian clock can occur not only when starting from an oscillatory state associated with cell proliferation (see Figs. 2–11), provided the autonomous period is in the adequate range, but also when starting from a stable steady state of the Cdk network, associated with cell quiescence. One among multiple ways to bring the oscillating Cdk network to a stable steady state is to raise the level of pRB at a given level of E2F. It is the balance between these two antagonistic factors that determines whether the network oscillates or not [14]. To see whether entrainment of the cell cycle can occur when the Cdk network is initially in a stable steady state, we start from the situation in Figs. 2–11 and increase moderately the level of pRB; the oscillations disappear and the system evolves to a stable steady state characterized by a low level of cyclin B/Cdk1. Upon coupling the cell cycle to the circadian clock through Wee1, circadian oscillations of cyclin B/Cdk1 are readily induced (Fig. 12A). The peak in cyclin B/Cdk1 again occurs at the end of the L phase, as it is dictated by the periodic variation of its inhibitor Wee1, which peaks at the end of the D phase. However, if we further increase the level of pRB, the Cdk network reaches a stable steady state that is now characterized by an even lower level of cyclin B/Cdk1. In such conditions, the same coupling to the circadian clock through Wee1 fails to entrain the cell cycle, because the brake exerted on the cell cycle by pRB is too strong (Fig. 12B).

Another way to bring the cell cycle to a quiescent state is to raise the level of the phosphatase Cdc25 that activates Cdk1. The Cdk network then evolves to a stable steady state characterized by a high level of cyclin B/Cdk1. In such conditions it is also possible to entrain the cell cycle through circadian variation of Wee1, as shown in Fig. 12C. The peak in cyclin B/Cdk1 again occurs at the end of the L phase, but it is larger and more similar to entrainment of the autonomous cell cycle oscillator than when a stable steady state is reached under high pRB levels (compare Fig. 12A with Fig. 12C and Fig. 3).

## Discussion

Experimental evidence gathered in unicellular and multicellular organisms indicates that the cell cycle is often gated by the circadian clock [2]–[7], [29], [30]. Several modes of coupling of the cell cycle to the circadian clock have recently been characterized in mammalian cells. First, the kinase Wee1, which inhibits the kinase Cdk1 and thereby impedes the G2/M transition, was shown to be induced by the transcriptional regulator BMAL1, which plays a central role in the network that generates circadian rhythms. Another Cdk inhibitor, p21, and cyclin E, are also controlled by BMAL1, via the proteins REV-ERBα and c-Myc, respectively. The purpose of this work was to investigate the dynamical consequences of these various modes of coupling of the cell cycle to the circadian clock. To address this issue we used a detailed computational model recently proposed for the Cdk network driving the mammalian cell cycle. Above a critical level of growth factor this network can display temporal self-organization in the form of spontaneous, sustained oscillations in the activity of the various Cdks that control the successive phases of the cell cycle [14]. Building on these results we investigated here the possibility of entraining the cell cycle by the circadian clock, which would give rise to circadian gating of cell division. Another goal of this study was to determine whether multiple modes of coupling facilitate entrainment. We further characterized the dynamics of the Cdk network when circadian entrainment fails to occur.

We first showed that the coupling of the cell cycle to the circadian clock through the kinase Wee1 is capable of entraining cell cycles with periods shorter or longer than 24 h, even though the domain of entrainment is asymmetric: the range of autonomous periods allowing for entrainment to the circadian clock is larger when the cell cycle period is smaller than 24 h. Whereas it is easy to see how an inhibitor such as Wee1 may slow down cell cycles of periods shorter than 24 h to bring them up to 24 h (Fig. 2C), it is less clear how an inhibitor of cell cycle progression can accelerate a cell cycle so as to bring its period from a value larger than 24 h down to a circadian value. As shown by the numerical simulations in Fig. 2D (see also Figs. 3B and 8B), such an entrainment can indeed occur because the coupling through Wee1 reduces the duration of the activity peak of the various cyclin/Cdk complexes, and thereby accelerates progression in the cell cycle. Note, however, that the half-width of the peaks in Cdk1 also reduces when passing from 20 h (Fig. 2A) to 24 h (Fig. 2C), much as in the case of Fig. 2D. This narrowing is due to the inhibition of Cdk1 by Wee1; the latter protein closely follows the profile of BMAL1, which begins to rise at the end of the L phase of the LD cycle and decreases at the beginning of the next L phase (see Fig. 7). This is also the reason why the peak of cyclin B/Cdk1, in the presence of entrainment by the circadian clock, occurs at the end of the light phase (Figs. 3, 8, 10, 12), which corresponds to the phase observed *in vivo* [23]. It would be interesting to test the prediction that entrainment of the cell cycle by the circadian clock is associated with a narrowing of the peaks in Cdk activity.

The model indicates that the lower the basal, circadian clock-independent rate of synthesis of the kinase Wee1, *v*_swee1_, the broader the domain of entrainment of the cell cycle by the circadian clock (compare Figs. 4A and B). Indeed, periods of the cell cycle larger than 24 h are more readily entrained at 24 h by the circadian clock when *v*_swee1_ is nil (Fig. 4B). In both panels of Fig. 4 the domain of entrainment is centered in a narrow band around 24 h at low strengths of coupling, and expands as the strength of coupling increases. Such *Arnold tongues*, often encountered in the synchronization of nonlinear oscillators by periodic forcing, are also observed here upon entrainment to 48 h when the autonomous period of the cell cycle is larger than 24 h. Interestingly, the model predicts that when the autonomous period roughly lies between 30 h and 40 h, a transition between entrainment to 48 h or 24 h can occur upon increasing only slightly the coupling strength when the domains for the two kinds of entrainment are very close. A similar transition is illustrated in Fig. 8 in the case of coupling through p21.

Gating of the cell cycle by the circadian clock may be achieved in different ways. Besides Wee1, other components of the cell cycle machinery such as p21 or cyclin E are controlled in a circadian manner [10]–[13]. Numerical simulations show that entrainment of the cell cycle can also occur through circadian variations in the Cdk inhibitor p21 (Fig. 8), or through circadian inhibition of the synthesis of cyclin E (Fig. 9C). Coupling can thus be achieved through promoting (in the case of Wee1, which is induced by BMAL1) or decreasing (in the case of p21, which is repressed by REV-ERBα, itself induced by BMAL1) a cell cycle inhibitor, or through inhibiting a cell cycle promotor (cyclin E, induced by c-Myc, which is repressed by BMAL1). The domain of entrainment does not enlarge when several or all three of these links between the cell cycle and the circadian clock operate simultaneously (see Fig. 9). What could then be the function of multiple coupling? The model suggests that such multiplicity does not facilitate entrainment but rather provides robustness through redundancy: when one mode of coupling ceases to operate, the others can take over and allow entrainment of the cell cycle by the circadian clock.

The model shows that the phases of the cell cycle are locked with respect to the circadian clock when entrainment to the latter occurs (compare Figs. 3, 8B and 10). In each of the domains of entrainment that we studied, the peak of cyclin B/Cdk1 (responsible for the entry into mitosis) appears at the end of the L phase of the light/dark cycle to which the circadian clock itself is entrained *in vivo*. The circadian proteins CLOCK-BMAL1 and REV-ERBα regulate the cell cycle proteins Wee1, p21, and cyclin E, which have different, sometimes antagonistic effects on the dynamics of the cell cycle. Thus, cyclin E and p21 are mainly active at the beginning of the cell cycle and control the G1/S transition, while Wee1 controls the G2/M transition. The model shows that the various modes of coupling may act in opposite ways: CLOCK-BMAL1 represses p21 and thereby activates Cdk2 and Cdk1, which promotes the S and M phases, but it also represses c-Myc and thereby reduces the expression of cyclin E and the activity of cyclin E/Cdk2, which effect inhibits the G1/S transition; CLOCK-BMAL1 further inhibits the G2/M transition by enhancing the expression of Wee1. This may explain why the combination of multiple modes of coupling fails to enlarge the domain of entrainment of the cell cycle by the circadian clock.

A reverse mode of coupling between the cell cycle and the circadian clock might arise from the temporary inhibition of transcription, in the course of mitosis [31]. Whether such an effect could pave the way for periodic forcing of the circadian clock by the cell cycle remains to be determined.

The model predicts that entrainment of the cell division cycle to a period of 24 h is not the only mode of dynamic behavior that can result from its coupling to the circadian clock. Indeed, if the autonomous period of the cell cycle is too small, entrainment fails and complex periodic behavior can emerge (Fig. 6A) as in point D in Fig. 4A. On the contrary, if the autonomous period of the cell cycle is too large, tetraploidy is observed (Fig. 6B) as in point E in Fig. 4A. When the coupling is too weak, irregular oscillatory behavior appears (Fig. 6C), as in point F in Fig. 4A. In contrast, if the strength of coupling is sufficiently large, the model predicts the emergence of endoreplication (Fig. 6D), as in point G in Fig. 4A; oscillations in Cdk2 then occur in the absence of periodic activation of Cdk1, i.e. multiple rounds of DNA replication take place without mitosis. The irregular oscillations in Fig. 6C correspond to nonautonomous chaotic behavior, because they result from the forcing of the Cdk network by the circadian clock. Chaotic oscillations in the Cdk network have also been observed in autonomous conditions as a result of the interplay between several oscillatory circuits present in the Cdk network [25].

Numerical simulations indicate that three peaks of Cdk1 per 24 h sometimes occur when the coupling strength is decreased, as shown in Fig. 7 established for points H and I in Fig. 4A. Can this be related to the experiments that show 3 peaks of mitosis per 24 h in fibroblasts cultures [6], or does the latter behavior result from a population effect, while here it is observed at the single cell level? In Fig. 7A large intervals separate triplets of peaks of Cdk1, which occur in the L phase, whereas in the experiments [6], the three peaks are roughly equidistant. In regard to the latter results, multiple peaks of the fraction of cells in S phase have been observed at the population level in an automaton model for the cell cycle, for an autonomous cell cycle length of 16 h [32]. This issue could be addressed by considering a population of cells, each of which would be governed by the same model for the cell cycle network, with some variations in the values of the kinetic parameters.

In regard to the effect of the coupling strength on cell cycle entrainment by the circadian clock the diagrams of Figs. 4 and 9 show that enhancing the coupling at first favors entrainment. However, when the coupling strength exceeds a critical value, entrainment fails to occur. This result is obtained for all three modes of coupling considered here. In the case of coupling through Wee1, entrainment failure stems from the fact that the level of this inhibitor rises at high coupling strengths, so that only minute oscillations in Cdk1 occur, accompanied by large-amplitude oscillations in Cdk2. This behavior corresponds to endoreplication (see Fig. 6D corresponding to point G in Fig. 4A).

For the three modes of coupling considered, we expressed the coupling through a circadian clock-controlled rate of mRNA synthesis of Wee1, p21, or cyclin E. Each of these rates is added to a constant, basal rate of mRNA synthesis independent from the circadian clock. The strength of coupling is then reflected by the relative magnitude of the circadian-dependent rate of synthesis. Alternative parameters could be used to measure coupling strength. Thus, we could use the activation constant *K*_aw_ in eq. [1] in Methods. This parameter measures the activation by BMAL1 of Wee1 expression, which increases as *K*_aw_ decreases. Similarly, for coupling through p21 or cyclin E we could use parameters *K*_ip21_ and *K*_ice_ in eqs. [3] and [5], respectively. These parameters measure the inhibitory effect exerted by the circadian clock on the expression of p21 and cyclin E. Much as for *K*_aw_, the coupling strength, i.e. the regulation exerted by the circadian clock on the cell cycle, increases as *K*_ip21_ and *K*_ice_ decrease.

Here, we coupled the cell cycle through the circadian clock by modifying the rate of synthesis of the coupling variables, i.e., the kinase Wee1, the Cdk inhibitor p21, or cyclin E. Experiments indeed show that the circadian clock proteins CLOCK-BMAL1 or REV-ERBα modulate the levels of these cell cycle proteins. Controlling the rate of synthesis of the latter proteins in a circadian manner appears to be the most natural and straightforward way to couple these two biological rhythms. Moreover, to test the predictions of the model, it might be easier to modify experimentally the rate of synthesis of the coupling variables, by resorting to specific deletion or circadian overexpression, rather than the activation or inhibition constants modulating the synthesis of these coupling variables. Our results nevertheless show that, with both modes of coupling, periods of the cell cycle smaller and larger than 24 h can be entrained to oscillate at a circadian period, and that domains of entrainment at 24 h or 48 h of the cell cycle by the circadian clock are also present when the inhibition or activation constants vary in a circadian manner (results not shown).

Outside the domain of entrainment, we can easily find conditions in the model where the circadian clock and the cell cycle oscillate in an independent manner. This behavior is encountered, obviously, in the absence of any coupling (see Figs. 2A and 2B) or when the coupling is too weak (e.g. in Fig. 5C corresponding to point C in Fig. 4A). Such a situation could apply to the experimental observation of circadian-independent cell cycling in immortalized fibroblasts [33]. Our results support the view that there might not be sufficient coupling between the cell cycle and the circadian clock in these cells. Perhaps the coupling exists in normal cells but is disconnected in such immortalized cells. One possible difference between cancer cells and normal cells could be the decreased capacity of the former to be entrained by the circadian clock [33].

The model predicts that domains of entrainment at 24 h or 48 h of the cell cycle by the circadian clock can both be observed. The model also shows that outside these domains of entrainment (i.e., when the coupling strength is not large enough or too large) the cell cycle and the circadian clock can operate as two dissociated rhythms, or complex dynamical behavior of the cell cycle might occur.

In this context it would be interesting to search experimentally for conditions in which the cell cycle might become temperature-compensated, as the circadian clock. The model raises the possibility that this may well occur, at least over a range of autonomous periods of the cell cycle, if the cell cycle were entrained by the circadian clock when the coupling strength is sufficiently large. The model suggests that as long as the autonomous period of the cell cycle, which diminishes when temperature increases, remains in the domain of entrainment by the circadian clock (see Figs. 4, 8 and 9), the cell cycle will acquire a circadian period and will become independent from temperature, i.e. temperature-compensated.

Does the existence of checkpoints in the cell cycle impinge on its capability of being entrained by the circadian clock? To address this issue we studied the effect of an activation of the DNA replication checkpoint on the circadian entrainment of the Cdk network (Fig. 11). Low activation of the checkpoint will not perturb cell proliferation (Fig. 11A), while higher activation can promote the occurrence of tetraploidy associated in the model with two peaks of Cdk2 per peak of Cdk1 (Fig. 11B). This result may account for the experimental observation that hyper-activation of the DNA replication checkpoint can lead to tetraploid cells [28]. Because the circadian clock can control ATR and Chk1 via the protein PER [34], [35], the DNA checkpoint controlled by the circadian clock could well represent an additional mode of coupling, which is also capable of eliciting by itself circadian entrainment of the cell cycle (results not shown).

We defined entrainment as the occurrence of one large-amplitude peak of Cdk2 and Cdk1 per 24 h or 48 h. As noted above, complex periodic oscillations may occur outside the domain of entrainment, with more than one peak of Cdk2 per peak of Cdk1. In these conditions, it is often possible to change such complex patterns of oscillations into a pattern of entrainment simply by enhancing the activation of the Cdk1 module. Thus, when the dynamical behavior of the Cdk network outside the domain of entrainment is characterized by an endoreplication process (see Fig. 11B) the model shows that increasing the rate of synthesis of cyclin A or cyclin B abolishes endoreplication and restores a correct entrainment of the cell cycle by the circadian clock characterized by one peak in both Cdk2 and Cdk1 per 24 h (results not shown).

**Figure 13:**
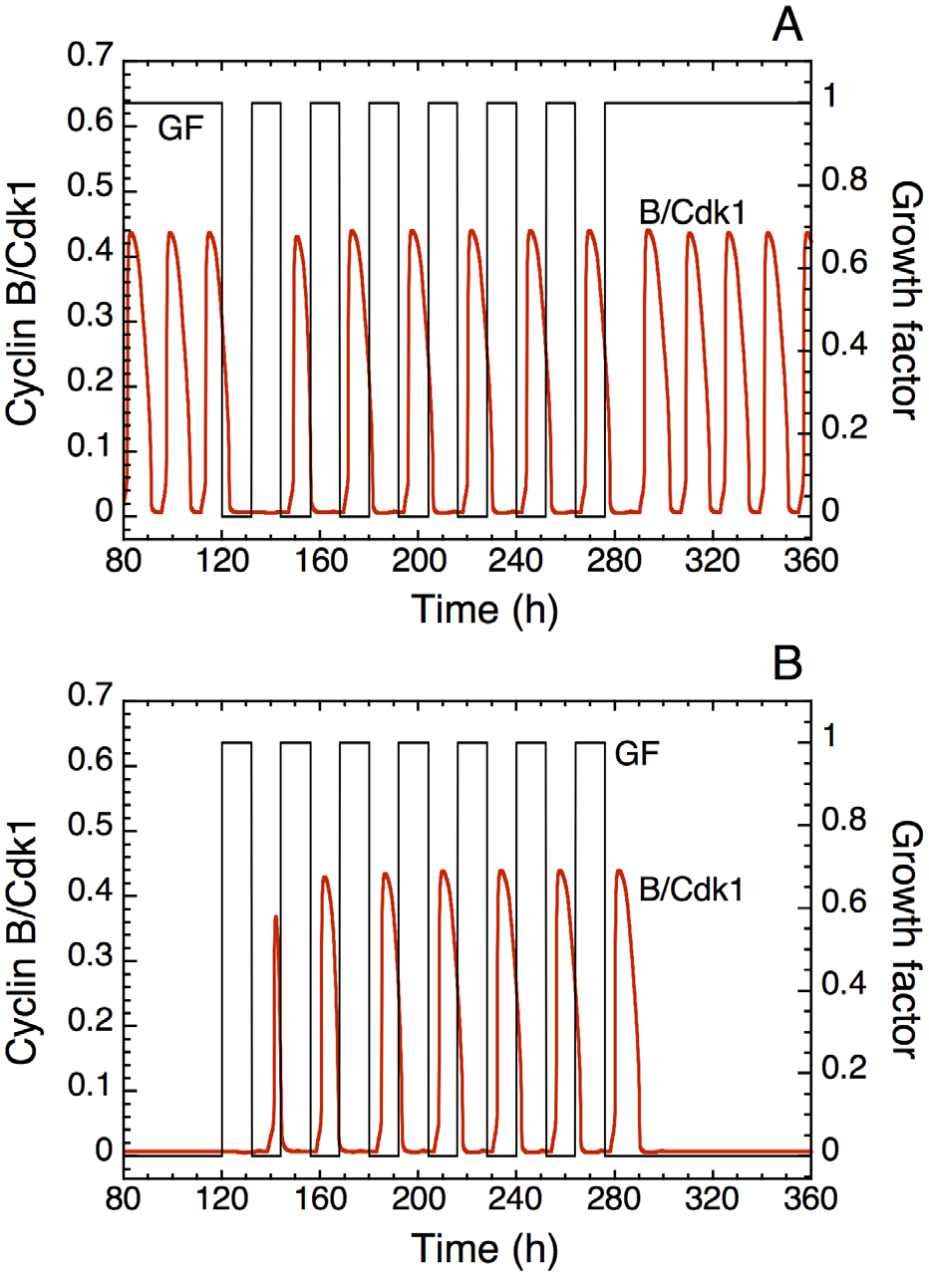
Entrainment of the cell cycle by a circadian variation of growth factor (GF). (A) In the continuous presence of GF at a constant value (GF = 1 µM), for t<120 h and t>280 h, cells proliferate with an autonomous period of 16 h. For 120 h<t<280 h, in the presence of a square wave variation of growth factor in which GF alternates every 12 h between the values 0 and 1 µM, the cell cycle readily entrains to the 24-h period of the square wave of GF. (B) Entrainment by the circadian variation of GF for t≥120 h also occurs when starting from a stable, quiescent state observed in the absence of GF [14]. Parameter values are listed in Table S2 of Ref. 14.

Circadian rhythms have been observed for many metabolic and cellular variables, including growth factors and growth factor receptors [15]–[17]. We assessed the possibility of entraining the cell cycle via the circadian variation in the level of growth factor. Numerical simulations predict (Fig. 13) that when the growth factor is delivered in the form of a square wave, the cell cycle can readily be entrained to oscillate at the same period.

**Figure 14:**
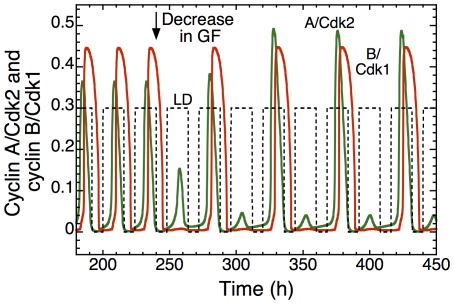
Growth-factor-induced switch in the pattern of entrainment of the cell cycle by the circadian clock. The curves show the time evolution of cyclin A/Cdk2 (green) and cyclin B/Cdk1 (red), as well as the LD cycle (dashed line). The Cdk network is coupled to the circadian clock through Wee1 and cyclin E, for μ = 0.028 —for this value of the coupling strength the domains of entrainment to 24 h or 48 h are very close to each other in the diagram of Fig. 9D. In these conditions a decrease in the level of the growth factor GF in t = 240 h (arrow) from 1 µM to 0.3 µM lengthens the autonomous period of the cell cycle from 21.6 h to 23.0 h. The rise in period associated with the decrease in GF results in shifting the period of the entrained cell cycle from 24 h to 48 h. Parameter values are as in Fig. 9D. The scaling parameter *eps* is equal to 15 (see Methods).

A specific prediction of the model in regard to the effect of growth factors pertains to the switch they may cause in the pattern of entrainment of the cell cycle by the circadian clock. When the cell cycle is coupled to the circadian clock, in appropriate conditions, a decrease in the level of growth factor GF could lead to an abrupt transition in cell cycle length, from 24 h to 48 h, as illustrated in Fig. 14. Such transition can also be achieved by other means, e.g. by decreasing the strength of coupling, as shown in Fig. 8. Let us stress that the switch from entrainment from 24 h to 48 h might not necessarily occur upon decreasing the level of GF. To achieve the abrupt doubling in cell cycle period, the system must shift from one domain of entrainment to the other. If this is not the case, the decrease in GF could either leave circadian entrainment unchanged, lead to independent oscillations in the cell cycle and the circadian clock, or result in the occurrence of complex patterns of periodic or aperiodic oscillations. If the switch in the pattern of entrainment from 24 h to 48 h were observed, it would provide a strong test of the view that the cell cycle behaves as a self-sustained oscillator capable of being entrained by the circadian clock.

**Figure 15:**
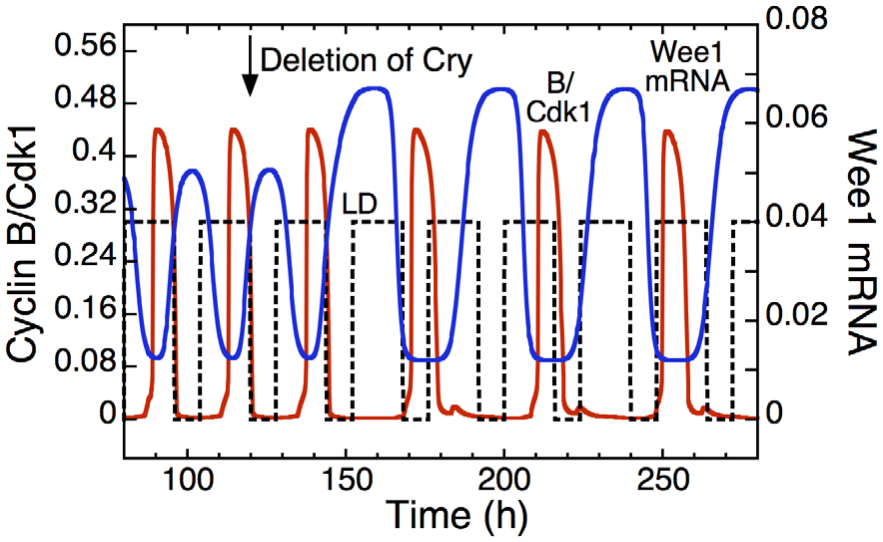
Dynamical behavior of the Cdk network in the model for coupling the cell cycle to the circadian clock upon deletion of the circadian clock protein Cry. The curves show the time evolution of cyclin B/Cdk1 (red curve) and Wee1 mRNA (blue curve) in the presence (t<120 h) or absence (t>120 h) of the clock protein Cry. For t<120 h, the cell cycle is entrained by the circadian clock via the kinase Wee1 to oscillate at 24 h (conditions correspond in Fig. 4A to an autonomous period of the cell cycle of 18 h and a coupling strength *v*_sw_, which is equal to 0.05 µM h^−1^). When Cry is deleted (t>120 h), the maximum level of Wee1 mRNA increases by about 35%, which fits with experimental observations (see [10]). The model also shows that in the absence of Cry, the circadian network ceases to oscillate while the cell cycle is slowed down; as a result of the increase in Wee1 the period of the cell cycle indeed passes from 24 h (its value upon entrainment) to nearly 40 h, which value is larger than its autonomous period in the absence of coupling, i.e. 18 h. Other parameter values are as in Fig. 4A.

The question arises as to whether the coupling strength required for entrainment is in the physiological range. In the model for the coupled cell cycle and circadian clock, a deletion of the circadian clock protein CRY leads to an overexpression of CLOCK-BMAL1, which raises the level of Wee1 mRNA by about 35% (see Fig. 15). This result fits with the experimental observation that the level of Wee1 mRNA increases between 30 and 40% in the Cry mutant (see Fig. 2 in [10]), suggesting that the coupling strength used in the model is likely of the order of that in the experiments. Cell cycle entrainment by the circadian clock through Wee1 is particularly robust as it occurs over four orders of magnitude of the coupling strength (see Fig. 4).

The model further shows that the increase in the level of Wee1 mRNA will slow down progression in the cell cycle in the situation considered in Fig. 15 where the period of the cell cycle passes from 24 h to nearly 40 h in the Cry mutant. This result holds with experimental observations showing that liver regeneration in Cry-deficient mice is much slower than in wild type [10].

The goal of this study was to investigate the modes of dynamical behavior resulting from the coupling of the cell cycle to the circadian clock. Do we need to address this question by resorting to a detailed model for the cell cycle or could we use a simplified description such as the skeleton, 5-variable model that we recently proposed for the Cdk network [36] driving the mammalian cell cycle? This much simpler model retains the major dynamical properties of the detailed 39-variable model [14] considered here. Because of its reduced number of variables, it appears to be well suited to study dynamical properties such as synchronization in a cell population. However, this reduced model is less adequate for studying entrainment of the cell cycle by the circadian clock because Wee1, p21 and cyclin E, which are explicitly included in the detailed model for the cell cycle considered here, are not included in the skeleton model in which many biochemical details were omitted in the reduction process. The detailed computational model for the mammalian cell cycle might be more cumbersome to use but it allows us to study entrainment through the various modes of coupling identified experimentally since these are incorporated explicitly into the model.

Many observations point to a close link between circadian rhythms and cancer [37]–[39]. Clinical studies have shown that the existence of robust circadian rhythms is positively correlated with the health status of cancer patients [40]. Perturbations of the circadian clock resulting from mutations of clock genes [12], [41] or from disruption of circadian pacemakers in animals [42], and evidence from epidemiological studies of shift workers in human populations indicate [43] that severe perturbations of circadian rhythms increase the risk of cancer. Entrainment of the cell cycle by the circadian clock appears to be of importance for normal cell proliferation and might be deregulated in cancer cells [44], [45]. Robust entrainment of the cell cycle by the circadian clock could play a protective role in physiological conditions.

## Methods

### Model for the Cdk network driving the mammalian cell cycle

The model for the Cdk network is governed by a system of 39 kinetic equations, which are listed as eqs. [1]–[39] in Supporting Information in Ref. 14 (http://www.pnas.org/content/106/51/21643/suppl/DCSupplemental) where the variables are defined in Table S1, while the definition and numerical value of the parameters are listed in Table S2. Five more kinetic equations listed in Ref. 14 as eqs. [40]–[44] are added when the DNA replication checkpoint involving the proteins Chk1 and ATR are incorporated into the model.

### Model for the mammalian circadian clock

The model for the mammalian circadian clock network is governed by a system of 19 kinetic equations, which are listed as eqs. ]1]–]19] in Supporting Information in Ref. 21 (http://www.pnas.org/content/100/12/7051/suppl/DC1) where parameter values are listed in the legend to Fig. 8, with *K*_ib_ = 1 nM.

### Coupling the cell cycle to the circadian clock through Wee1

To describe the coupling of the cell cycle to the circadian clock via the kinase Wee1 we incorporate in the model for the Cdk network an additional kinetic equation for the mRNA of the kinase Wee1, which includes the induction by CLOCK-BMAL1 of Wee1 expression. The equations [1] and [2] describing the coupling of the cell cycle to the circadian clock through the activation of the transcription of Wee1 mRNA by CLOCK-BMAL1 are:
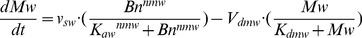
(1)

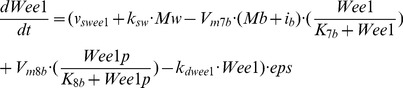
(2)These equations replace eq. [38] in the supporting Information in Ref. 14. Here, eq. [1] describes the time evolution of the Wee1 mRNA positively regulated by the circadian clock complex, CLOCK-BMAL1 (*Bn*), with an activation constant 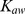, while eq. [2] shows the time evolution of the kinase Wee1. The synthesis of this protein kinase combines two terms: the basal rate of Wee1 synthesis (*v*_swee1_) is independent from the circadian clock, while the second term, (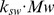), reflects Wee1 synthesis at a rate proportional to the Wee1 mRNA controlled by CLOCK-BMAL1 in a circadian manner. The total amount of Wee1 mRNA can therefore be viewed as the sum of two terms, (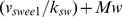), which are respectively independent from and dependent on the circadian clock. The coupling strength is expressed by parameter *v*_sw_, which measures the magnitude of the Wee1 mRNA synthesized under control by the circadian clock. An alternative measure of coupling strength is provided by the parameter *K*_aw_, which characterizes the activation of Wee1 expression by CLOCK-BMAL1; the strength of this coupling to the circadian clock increases as *K*_aw_ progressively decreases.

### Coupling the cell cycle to the circadian clock through p21

To incorporate this mode of coupling into the model, we consider that the protein REV-ERBα (*Rn*), which is a variable of the model for the mammalian circadian clock, can repress the synthesis of p21 (and its homologue p27) mRNA. The temporal evolution of the circadian clock-dependent p21 mRNA (*Mp21*) and of the total concentration of the p21/p27 protein is described by eqs. [3] and [4], which replace eq. [24] in Ref. 14:
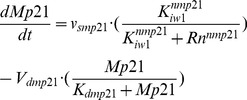
(3)

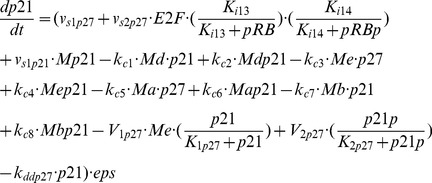
(4)Two main terms regulate the synthesis of p21 in eq. [4]: one does not depend on the circadian clock and contains a basal, constant component as well as a component activated by E2F and inhibited by pRB, 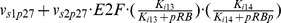, while the other component depends on the circadian clock, 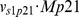. If the inhibition by REV-ERBα is removed in eq. [3], the levels of p21 mRNA and p21 protein increase monotonically until a stable steady state is reached. This fits with the observation that when BMAL1, which promotes the expression of REV-ERBα, is mutated, the level of p21 rises and circadian oscillations disappear [11].

### Coupling the cell cycle to the circadian clock through cyclin E

BMAL1 indirectly represses the expression of the cyclin E gene. Indeed, BMAL1 inhibits c-Myc, which itself induces the expression of cyclin E. The temporal evolution of the cyclin E mRNA (*Mce*) controlled by the circadian clock and of the cyclin E protein (*Ce*) is described by eqs. [5] and [6], respectively. These equations replace eq. [13] in Ref. 14. For simplicity we consider that the synthesis of cyclin E mRNA is directly repressed by CLOCK/BMAL1, while the synthesis of the cyclin E protein, *Ce*, is described in eq. [6] by two terms: the first, activated by E2F and inhibited by pRB, does not depend on the circadian clock, 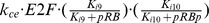, and the second depends on it through CLOCK-BMAL1, 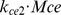:
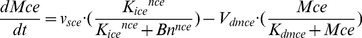
(5)

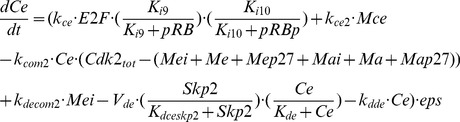
(6)


### Coupling the cell cycle to the circadian clock through Wee1, p21 and cyclin E

The coupling of the cell cycle by the circadian clock through Wee1, p21 and cyclin E is achieved by multiplying by the same dimensionless parameter μ the parameters controlled by the circadian clock, i.e. the rate of synthesis of Wee1 mRNA, *v*_sw_, the rate of synthesis of p21 mRNA, *v*_smp21_, and the rate of synthesis of cyclin E mRNA, *v*_sce_ (see eqs. [1], [3], and [5]). These rates of synthesis are put equal to 1 µM h^−1^. The multiplicative factor μ then measures the strength of coupling in Fig. 9.
